# Peptidoglycan analysis reveals that synergistic deacetylase activity in vegetative *Clostridium difficile* impacts the host response

**DOI:** 10.1074/jbc.RA119.012442

**Published:** 2021-01-13

**Authors:** Héloise Coullon, Aline Rifflet, Richard Wheeler, Claire Janoir, Ivo G. Boneca, Thomas Candela

**Affiliations:** 1 Université Paris-Saclay, INRAE, AgroParisTech, Université Paris-Saclay, Jouy-en-Josas, France; 2 Institut Pasteur, Unité Biologie et Génétique de la Paroi Bactérienne, Paris, France; 3 INSERM, Équipe Avenir, Paris; 4 CNRS, UMR 2001 “Microbiologie Intégrative et Moléculaire,” Paris, France

**Keywords:** Clostridium difficile, peptidoglycan, N-deacetylase, lysozyme, virulence, cell wall, bacteria, bacterial pathogenesis, bacterial metabolism

## Abstract

*Clostridium difficile* is an anaerobic and spore-forming bacterium responsible for 15–25% of postantibiotic diarrhea and 95% of pseudomembranous colitis. Peptidoglycan is a crucial element of the bacterial cell wall that is exposed to the host, making it an important target for the innate immune system. The *C. difficile* peptidoglycan is largely *N*-deacetylated on its glucosamine (93% of muropeptides) through the activity of enzymes known as *N*-deacetylases, and this *N*-deacetylation modulates host–pathogen interactions, such as resistance to the bacteriolytic activity of lysozyme, virulence, and host innate immune responses. *C. difficile* genome analysis showed that 12 genes potentially encode *N*-deacetylases; however, which of these *N*-deacetylases are involved in peptidoglycan *N-*deacetylation remains unknown. Here, we report the enzymes responsible for peptidoglycan *N*-deacetylation and their respective regulation. Through peptidoglycan analysis of several mutants, we found that the *N-*deacetylases PdaV and PgdA act in synergy. Together they are responsible for the high level of peptidoglycan *N*-deacetylation in *C. difficile* and the consequent resistance to lysozyme. We also characterized a third enzyme, PgdB, as a glucosamine *N*-deacetylase. However, its impact on *N*-deacetylation and lysozyme resistance is limited, and its physiological role remains to be dissected. Finally, given the influence of peptidoglycan *N*-deacetylation on host defense against pathogens, we investigated the virulence and colonization ability of the mutants. Unlike what has been shown in other pathogenic bacteria, a lack of *N*-deacetylation in *C. difficile* is not linked to a decrease in virulence.

Lysozyme is one of the most frequent and most abundant molecules of the innate immune system, and it is found in many bodily fluids, such as tears or saliva, as well as tissues, including the intestinal tract (1, 2). The bactericidal activity of lysozyme is linked to two distinct mechanisms. The most well-known mechanism is the lytic activity: lysozyme can act as an *N*-acetylmuramidase and hydrolyze the β-1,4-linkage between the *N*-acetylmuramic acid residue and the GlcNAc residue of peptidoglycan muropeptide chains (1). The second mechanism relies on the action of lysozyme as a cationic antimicrobial peptide, and it was suggested that lysozyme could insert into negatively charged membranes and form pores, which induces membrane alteration and cell death (1).

Bacteria have several mechanisms to protect themselves from lysozyme, through modification of the peptidoglycan structure or alteration of the cell-surface charge (3). In addition, it has also been shown that bacteria such as *Escherichia coli* or *Pseudomonas aeruginosa* can produce proteinaceous lysozyme inhibitors (reviewed in Ref. 4). Modification of peptidoglycan by *N*-deacetylation has been regarded as one of the hallmarks of lysozyme resistance in many bacterial species. *N*-Deacetylation is a chemical reaction involving the removal of an acetyl group from the acetamido group of GlcNAc or *N-*acetylmuramic acid moieties. Enzymes responsible for such reactions are called *N*-deacetylases, and they can target several bacterial components in addition to peptidoglycan, such as the spore cortex (5). *N*-Deacetylases targeting peptidoglycan typically target the GlcNAc of the muropeptides. The first bacterial peptidoglycan *N*-deacetylase was identified in *Streptococcus pneumoniae* and called PgdA for “peptidoglycan deacetylase A” (6). Since then, many *N*-deacetylases have been studied in organisms such as *Listeria monocytogenes*, *Bacillus anthracis*, or *Enterococcus faecalis* (7, 8, 9). Through these studies, *N*-deacetylation has been linked to several host–pathogen mechanisms. Indeed, *N*-deacetylation of *L. monocytogenes* muropeptides has been shown to decrease recognition by the NOD immune receptors, with additional roles in intracellular survival, and ultimately in virulence (9, 10). In *L. monocytogenes*, the Δ*pgdA* mutant shows a virulence defect similar to the defect observed for the listeriolysin O mutant, which is one of the major virulence factors of this bacterium (9). *N*-Deacetylases have also been characterized as virulence factors in other organisms. In *Streptococcus suis*, the PgdA *N*-deacetylase is involved in phagocytosis evasion and cytokine response regulation (11, 12). In *B. anthracis*, two *N*-deacetylases are involved in the anchoring of the γ-glutamyl capsule, a structure with a central role in virulence (13).

*Clostridium difficile* is a Gram-positive spore-forming, toxin-producing anaerobic bacterium that can colonize the intestinal tract of humans and other animals (14, 15). It is a major nosocomial pathogen, and *C. difficile* infection (CDI) can lead to a spectrum of clinical signs, ranging from simple self-limiting diarrhea to life-threatening pseudomembranous colitis. Several risk factors have been described for CDI, including antibiotic exposure and compromised immune systems (16). In healthcare settings, *C. difficile* is considered the leading cause of antibiotic-associated diarrhea in adults (17, 18, 19). The peptidoglycan of *C. difficile* was described by Peltier *et al.* in 2011 (20) as having highly unique characteristics. The pentapeptide stem is composed of l-alanine, γ-d-glutamate, *meso*-diaminopimelic acid, d-alanine, and d-alanine (l-Ala/d-Glu/*meso*DAP/d-Ala–d-Ala) (20). In the peptidoglycan, the majority of muropeptides are tetrapeptides (65% of all muropeptides) and tripeptides (27% of muropeptides). Dimers represent 56% of muropeptides, and the peptidoglycan has a global cross-linking index of 33.8%. Moreover, 75% of the cross-links occur between two *meso*DAP residues (3–3 cross-link) instead of the traditional *meso*DAP–d-Ala (4–3 cross-link), which only represent 25% of cross-links. Additionally, the peptidoglycan has been associated with a strikingly high level of *N*-deacetylation. Indeed, 93% of all muropeptides are *N*-deacetylated on the GlcNAc residues. Consistent with this observation, *C. difficile* is resistant to lysozyme with MIC values of >1 mg/ml (21).

Although there are 10 potential surface peptidoglycan *N*-deacetylases predicted in the genome of *C. difficile*, they remain poorly studied so far. The first *N*-deacetylase described in *C. difficile*, PdaV, was studied by Ho *et al.* (21) in 2014, focusing on the regulation of lysozyme resistance by the sigma V (*csfV*, σ^V^) transcription factor. PdaV is a membrane associated peptidoglycan *N*-deacetylase shown to be strongly expressed in the presence of lysozyme, through the σ^V^ factor. Its gene is the first of the *csfV* operon. The peptidoglycan analysis published for the *csfV* mutant shows a decrease of *N*-deacetylation, and introducing PdaV in a *csfV* mutant induces *N*-deacetylation of approximatively 5% of muropeptides. These results suggested that PdaV plays a role in peptidoglycan *N*-deacetylation after exposure to lysozyme. However, it would appear as though PdaV alone is not responsible for the high native level of peptidoglycan *N*-deacetylation in *C. difficile*. Recently, we also characterized PdaA1 and PdaA2, two *N*-deacetylases involved in spore peptidoglycan modification (5).

The aim of this study was to characterize the *N*-deacetylases involved in the high level of peptidoglycan *N*-deacetylation. Here, we report the characterization of CD630_15220, renamed PgdA, as a major *N*-deacetylase of *C. difficile*, as well as the *N*-deacetylase CD630_32570, renamed PgdB, and we address their complex interplay. Given the impact of *N*-deacetylation in the host–pathogen relationship, the respective roles of these *N*-deacetylases in virulence and colonization have been investigated as well.

## Results

### Identification of the N-deacetylase candidates

The *C. difficile N*-deacetylase PdaV (CD630_15560) has been associated with *N*-deacetylation of approximatively 5% of muropeptides after induction by lysozyme (21). To identify which of the putative *N*-deacetylases were involved in peptidoglycan *N*-deacetylation, a ClustalOmega analysis of known *N*-deacetylases was performed (22). In the identity matrix obtained, the *C. difficile* putative *N*-deacetylases CD630_15220 and CD630_32570 were the closest to PdaV, with sequence identities of 24.9 and 43.8% respectively (Table S1) (22). Moreover, these three *N*-deacetylases share some degree of similarity with known peptidoglycan *N*-deacetylases, and most notably CD630_15220 with PgdA from *E. faecalis* (33.8% identity) (7, 22). Using the bioinformatics tool PredLipo, we found that CD630_15220 and CD630_15560 both have a potential signal peptide and transmembrane domains, suggesting that they may be surface-associated, whereas CD630_32570 does not have a signal peptide but harbors potential transmembrane domains (23). Consistent with this prediction, CD630_15220 has been detected in the surface proteome of *C. difficile* biofilm (24). Taking into consideration the results presented in this study, we propose to name *CD630_15220* as *pgdA* and *CD630_32570* as *pgdB*.

### C. difficile has two N-deacetylases involved in lysozyme resistance

**Figure 1:**
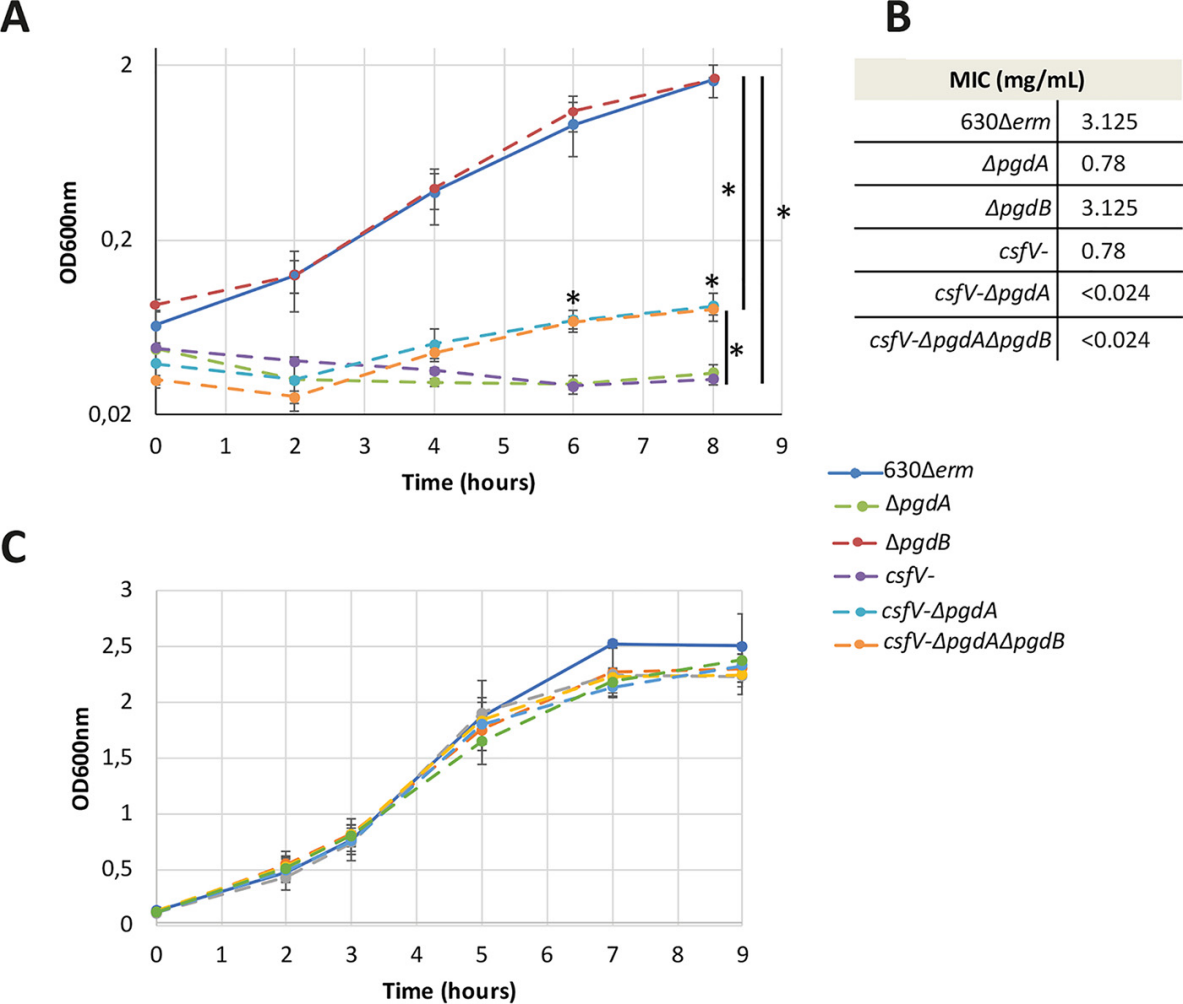
***C. difficile* has two *N*-deacetylases involved in lysozyme resistance.***A*, growth curves of the parental strain (*solid line*) and mutants (*dotted lines*) in BHISG supplemented with 1 mg/ml lysozyme. Δ*CD630_15220* (Δ*pgdA*) is in *green*, Δ*CD630_32570* (Δ*pgdB*) is in *red*, *csfV-* (with no expression of *pdaV*) is in *purple*, *csfV-*Δ*pgdA* is in *cyan*, and *csfV-*Δ*pgdA*Δ*pgdB* is in *orange*. *B*, the MIC of lysozyme was determined for the parental strain and each mutant. *C*, growth curve of the parental strain (*solid line*) and mutants (*dotted lines*) in BHISG without lysozyme. The results are represented as the means and standard deviations of at least three biological replicates. *, Student's *t* test, *p* < 0.05.

To test the potential involvement of these *N*-deacetylases in peptidoglycan *N*-deacetylation, knockout mutants were built for *pgdA* (CD630_15220) and *pgdB* (CD630_32570). The *csfV*- mutant (25), with no expression of *pdaV* (CD630_15560), was incorporated in our study. All the mutants had growth comparable with the parental strain (630Δ*erm*); however, the Δ*pgdB* mutant was the only one showing a growth similar to the parental strain in the presence of lysozyme (Fig. 1*A*). In comparison, optical density of the Δ*pgdA* and *csfV*- mutants decreased during the first 6 h in the presence of lysozyme and barely reached *A*_600nm_ = 0.03 after 8 h, suggesting that both PgdA and PdaV are involved in lysozyme resistance. To investigate potential redundancy, we built the *csfV*-Δ*pgdA* double mutant and *csfV-*Δ*pgdA* Δ*pgdB* triple mutant. The double and triple mutants showed a strong growth defect in the presence of lysozyme compared with the parental strain (Fig. 1*A*, Student's *t* test, *p* < 0.05) but show an increased growth compared with the Δ*pgdA* and *csfV*- mutant. This suggests that the growth defects of the double and triple mutants are less striking than the defects observed for the Δ*pgdA* and *csfV-* mutants (Fig. 1*A*, Student's *t* test, *p* < 0.05). In comparison, all the mutant strains showed a growth similar to the parental strain in media without lysozyme (Fig. 1*C*), suggesting that there is no growth defect in either of the mutants. Morphology of each mutant was assessed after 6 and 24 h of growth in the presence of lysozyme (Fig. S1). After 24 h growth, the Δ*pgdB*, Δ*pgdA*, and *csfV-* mutant strains show slightly curved medium sized bacilli similar to the bacilli of the parental strain (analyzed on at least 90 bacilli). In comparison, examination of the double and triple mutant strain samples reveals only numerous circular forms and no bacilli (56 and 51 round forms, respectively, in 10 fields of view). Although less abundant, these forms are already present for the double and triple mutants after 6 h of growth (Fig. S1). In these conditions, these results suggest that the increased optical density obtained for the double and triple mutant strains in lysozyme-supplemented medium is the result of an abnormal morphology development resembling an L-form shape.

To quantify the lysozyme sensitivity of each strain, we determined their respective MICs for hen egg-white lysozyme (Fig. 1*B*). Consistent with the growth curve, the Δ*pgdA* and *csfV-* mutants show a similar decrease in MIC for hen egg-white lysozyme (Fig. 1*B*, 0.78 mg/ml compared with 3.125 mg/ml for the parental strain, Student's *t* test, *p* < 0.05). Moreover, the Δ*pgdB* mutant has a lysozyme resistance similar to the parental strain. However, the double and triple mutant strains have a highly decreased MIC compared with the simple mutants (Fig. 1*B*, <0.024 mg/ml, respectively; Student's *t* test, *p* < 0.05). These results suggest that PgdA and PdaV are involved in lysozyme resistance in *C. difficile*.

### Lysozyme sensitivity of the ΔpgdA mutant can be rescued by expression of either pgdA or CD630_32570

**Figure 2:**
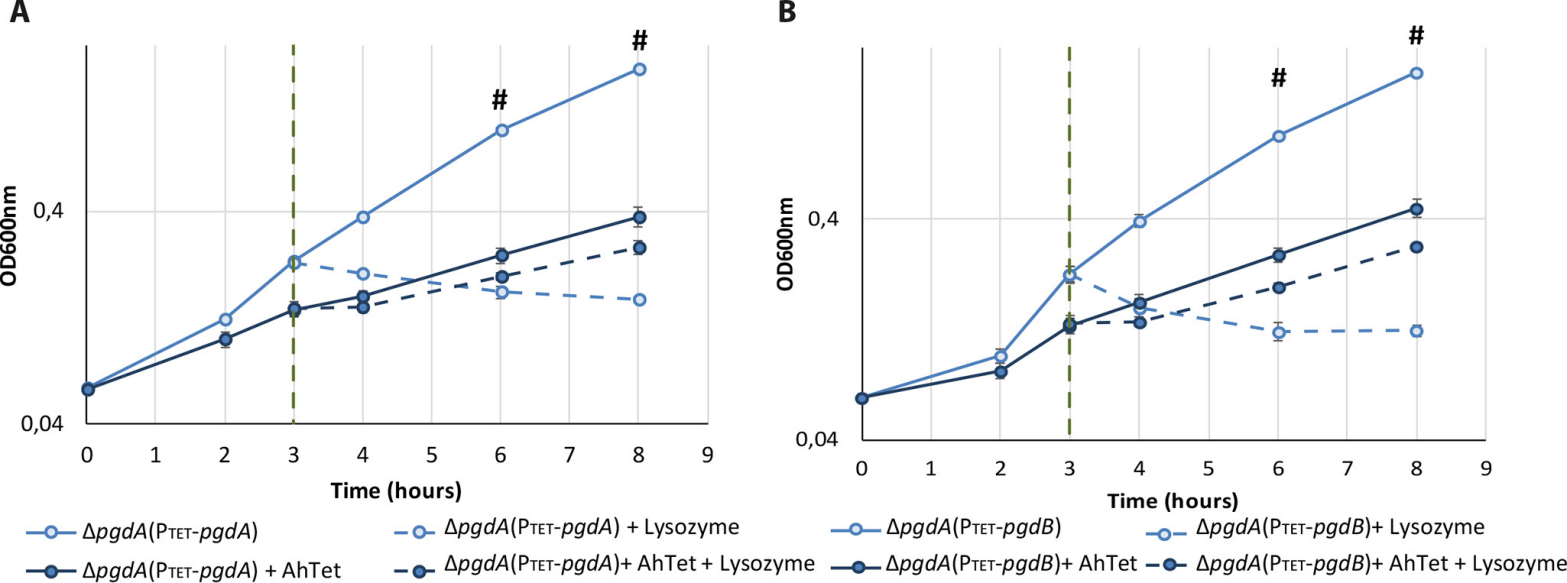
**Complementation using *pgdA* or *pgdB* restores lysozyme resistance in the Δ*pgdA* mutant.***A*, growth curve of the Δ*pgdA* mutant carrying the pCH71 plasmid for *pgdAB* complementation (named P_TET_-*pgdA* in the figure): the *light blue growth curves* represent cultures for which the complementation using *pgdA* was not induced by anhydrous tetracycline, whereas the *dark blue curves* indicate cultures in which *pgdA* was expressed by addition of anhydrous tetracycline. *B*, growth curve of the Δ*pgdA* mutant carrying the pCH70 plasmid for *pgdB* expression (named P_TET_-*pgdB* in the figure). The *light blue growth curves* with represent cultures where *pgdB* expression was not induced by anhydrous tetracycline, whereas the *dark blue curves* indicate cultures with anhydrous tetracycline in which *pgdB* expression was induced. Lysozyme is added 3 h after inoculation at 1 mg/ml, represented on the graph by the *vertical dotted line*, and cultures for which lysozyme is added in the medium are indicated by *dashed lines*. The results are represented as the means and standard deviations of at least three biological replicates. #, Student's *t* test, *p* < 0.05.

As discussed above, the *csfV-* mutant has been investigated previously (25), including *pdaV* complementation into the *csfV-* mutant. We therefore focused on lysozyme phenotype for the Δ*pgdA* mutant strain harboring either the P_TET_ inducible *pgdA* complementation plasmid (Fig. 2, Δ*pgdA*(P_TET_-*pgdA*)) or P_TET_ inducible *pgdB* expression (Fig. 2, Δ*pgdA*(P_TET_-*pgdB*)). In both experiments, when expression is not induced by anhydrous tetracycline, addition of lysozyme in the medium induces an interruption of growth, followed by cell lysis (Fig. 2, Student's *t* test, *p* < 0.005). In comparison, when Δ*pgdA*(P_TET_-*pgdA*) complementation is induced by anhydrous tetracycline, the addition of lysozyme only induces a minor decrease in the growth kinetics (Fig. 2, Student's *t* test, *p* > 0.005). These results suggest that expression of *pgdA* in the Δ*pgdA* mutant restores lysozyme resistance.

In the same way, when strains are grown with anhydrous tetracycline, the addition of lysozyme in the medium only induces a slight decrease in the growth rate of the Δ*pgdA*(P_TET_-*pgdB*) strain, similar to that seen for Δ*pgdA*(P_TET_-*pgdA*) complementation. These results indicate that the lysozyme sensitivity of the Δ*pgdA* mutant can be rescued by *pgdB* expression, which suggests that PgdB might be able to deacetylate peptidoglycan.

### PdaV and PgdA act in synergy to deacetylate the peptidoglycan GlcNAc

**Figure 3:**
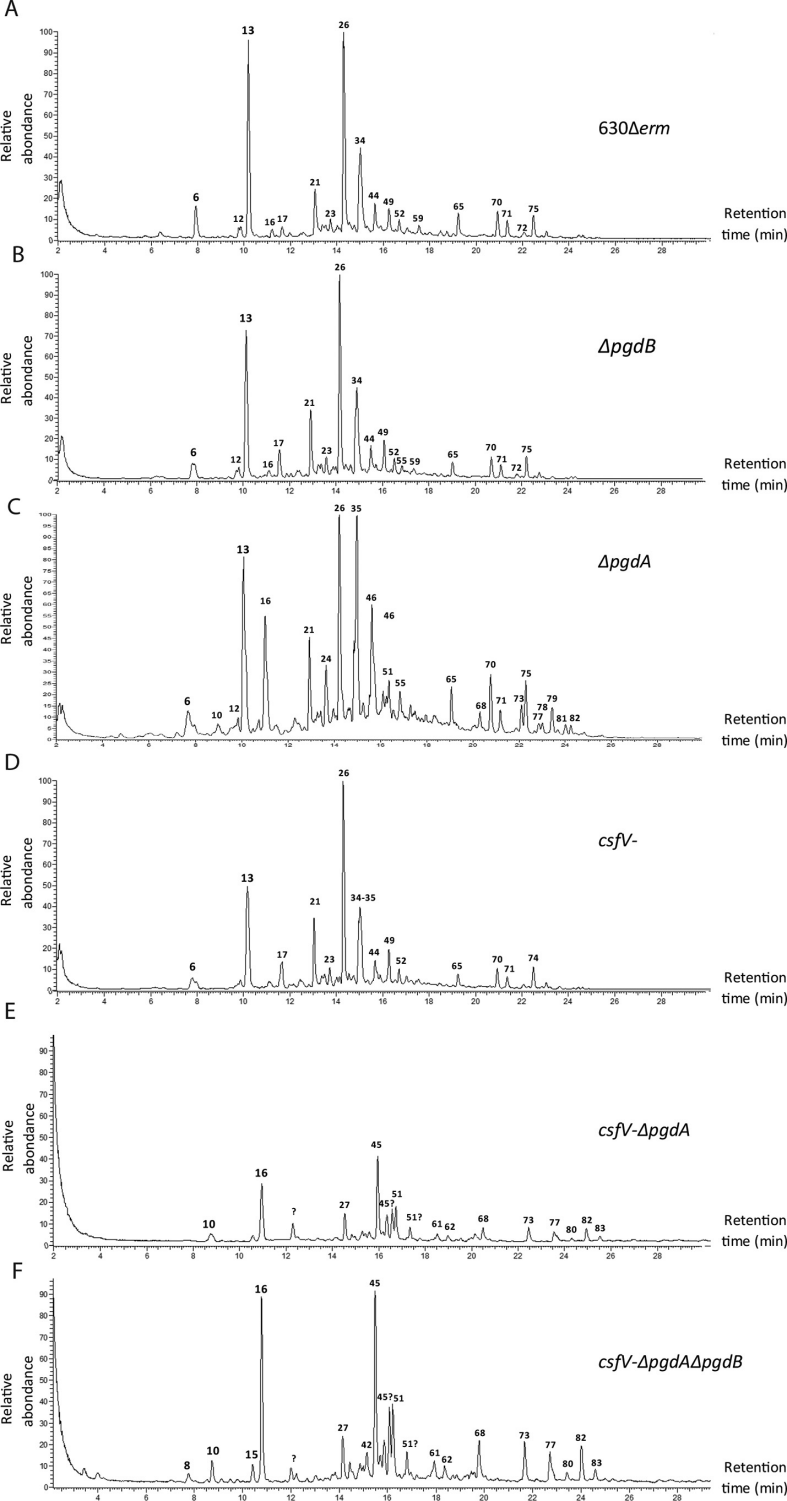
**Muropeptide analysis of the vegetative cell peptidoglycan by high-resolution mass spectrometry–coupled ultra-high-performance liquid chromatography.** Muropeptide composition was analyzed for the 630Δ*erm* parental strain (*A*) and the Δ*pgdB* (*B*), Δ*pgdA* (*C*), *csfV-* (*D*), *csfV-*Δ*pgdA* (*E*), and *csfV-*Δ*pgdA*Δ*pgdB* (*F*) mutants. *Numerical peaks* refer to the muropeptides listed in Table S2.

The mutant strains were grown in medium without lysozyme, and the peptidoglycan composition of all the mutant strains was analyzed and compared with that of the parental strain (Table S2, Fig. 3, and Fig. S2). In our analysis, we found a high level of *N*-deacetylation of the peptidoglycan for the parental strain, as already described previously (97.02% in our study, 93% in Ref. 20, and 88.6% in Ref. 21). However, we also detected additional new muropeptides present in low amounts (Table S2). Of note, we identified new muropeptides with putative structures consistent with amino acids such as lysine, valine, phenylalanine, or a modified alanine. Altogether, we identified over 40 peaks that have not been described in *C. difficile* before. In our mutant analysis, we found a similar level of *N*-deacetylation between the Δ*pgdB* and *csfV-* mutants as compared with the WT (94.4 and 92%, respectively; Table 1). However, the Δ*pgdA* mutant has a decrease in *N*-deacetylated peptidoglycan (73.7%, Student's *t* test, *p* < 0.05). Given the *N*-deacetylation level remaining for the single mutants, we analyzed the double mutant *csfV-*Δ*pgdA* and triple mutant *csfV-*Δ*pgdA*Δ*pgdB*, both of which had a drastic decrease in peptidoglycan *N*-deacetylation (7.4 and 4.6%, Student's *t* test, *p* < 0.05) compared with the parental strain. This result suggests that although PgdA seems to have a higher impact on its own, PdaV and PgdA act in synergy to deacetylate the GlcNAc of the peptidoglycan, and together they are responsible for the high level of peptidoglycan *N*-deacetylation in *C. difficile*. Complementation of the Δ*pgdA* mutant restores the peptidoglycan *N*-deacetylation level to a value close to the parental strain (Table 1), whereas expression of *pgdB* from the P_TET_ promoter in the Δ*pgdA* background did not show a significant change in peptidoglycan *N*-deacetylation compared with the Δ*pgdA* mutant carrying the empty plasmid (83.9 and 82.9%, respectively).

**Table 1:** Peptidoglycan *N*-deacetylation levels *N*-Deacetylation levels of the peptidoglycan analysis of three independent biological replicates, processed as detailed in Fig. 5 and Table S2. The *N*-deacetylation was determined extracted ion chromatogram and defined as the proportion of the peak area of muropeptides *N*-deacetylated on the glucosamine divided by the total muropeptides area × 100. The results are represented as the means and standard deviations of three biological replicates.

Strain	*N*-Deacetylation
	%
630Δ*erm*	97.5 ± 0.7
Δ*pgdB*	94.4 ± 0.7
Δ*pgdA*	73.7 ± 1.4^a^
*csfV-*	92.0 ± 0.3
*csfV*::*erm* Δ*pgdA*	7.4 ± 1.2^a^
*csfV*::*erm* Δ*pgdA* Δ*pgdB*	4.6 ± 0.6^a^
Δ*pgdA*(pRPF185)	82.9 ± 1.1
Δ*pgdA*(P_TET_-*pgdB*)	83.4 ± 2.3
Δ*pgdA*(P_TET_-*pgdA*)	87.8 ± 1.3^a^


a Student's *t* test, *p* < 0.05.

### PgdA and PdaV are lysozyme-induced N-deacetylases with interconnected yet different regulation patterns

**Figure 4:**
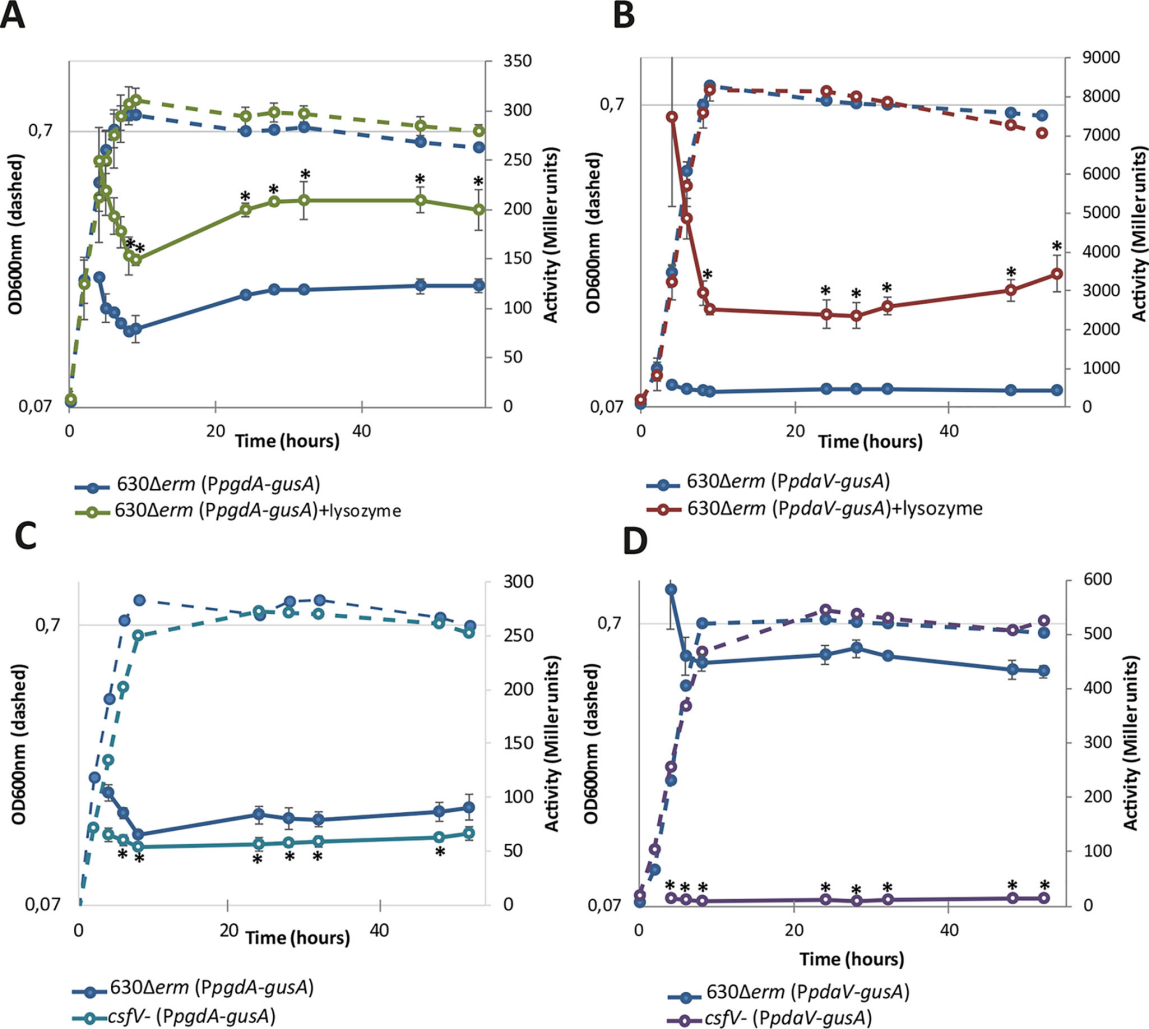
**β-Glucuronidase activity from P*pgdA*-*gusA* and P*csfV*-*gusA* fusions.** We studied the impact of lysozyme on *pgdA* and *pdaV* expression (*A* and *B*), as well as the impact of σ^V^ (*C* and *D*) through the β-glucuronidase activity from P*pgdA*-*gusA* (pCH32) and P*pdaV*-*gusA* (pCH56). Expression in the parental strain in medium without lysozyme is represented as *dark blue curves*. Expression of *pgdA* (P*pgdA*-*gusA*) or *pdaV* (P*pdaV*-*gusA*) in the parental strain with lysozyme added in the medium is represented respectively in *cyan* in *C* and in *dark blue* in *D*. The expression of *pgdA* (P*pgdA*-*gusA*) and of *pdaV* (P*pdaV*-*gusA*) in the *csfV-* mutant are represented respectively in *cyan* in *C* and in *dark blue* in *D*. The growth curve is shown in *dotted lines*, and the β-glucuronidase activity is shown in *solid lines*. The results are represented as the means and standard deviations of at least three biological replicates. *, Student's *t* test, *p* < 0.05.

The PdaV *N*-deacetylase has been previously described by Ho *et al.* (21) as the only lysozyme-induced *N*-deacetylase (21). However, our peptidoglycan analysis suggests that PdaV might even be involved in peptidoglycan *N*-deacetylation without lysozyme. The expression patterns of *pgdA* and *pdaV* were assessed in the WT strain: in these assays, PgdA and PdaV were found to be expressed throughout the growth curve, with a GusA*A* activity of 100 and 400 Miller units, respectively (Fig. 4, *A* and *B*). Adding lysozyme in the growth medium induced a 14–18-fold increase in P*_pdaV_*-*gusA* activity (Fig. 4*B*, Student's *t* test, *p* < 0.005), as well as a 2-fold increase in P*_pgdA_*-*gusA* activity (Fig. 4*A*, Student's *t* test, *p* < 0.005). These results suggest that PgdA and PdaV are expressed constitutively and that their expression is also induced by lysozyme.

Because lysozyme regulation in *C. difficile* involves the extracytoplasmic function sigma factor σ^V^, the expression of *pgdA* and *pdaV* was assessed in the *csfV-* mutant and compared with their respective expression in the parental strain. As expected, whereas P*_pdaV_-gusA* activity reached 600 Miller units in the parental strain in the exponential phase, *pdaV* is not expressed in the *csfV-* mutant strain (Fig. 4*D*, *violet curve*). In comparison, the P*_pgdA_-gusA* activity showed a slight but statistically significant decrease in the *csfV-* mutant compared with the parental strain (Fig. 4*C*). These results suggest that the *pdaV* expression is entirely dependent on σ^V^, even without lysozyme added in the medium. Moreover, the results suggests that the σ^V^ factor also influences *pgdA* expression, but to a much lower extent. Taken together, these results suggest that both *N*-deacetylases share some common regulatory mechanisms, but the respective impact on their expression varies.

### PgdB is likely not involved in vegetative peptidoglycan N-deacetylation

**Figure 5:**
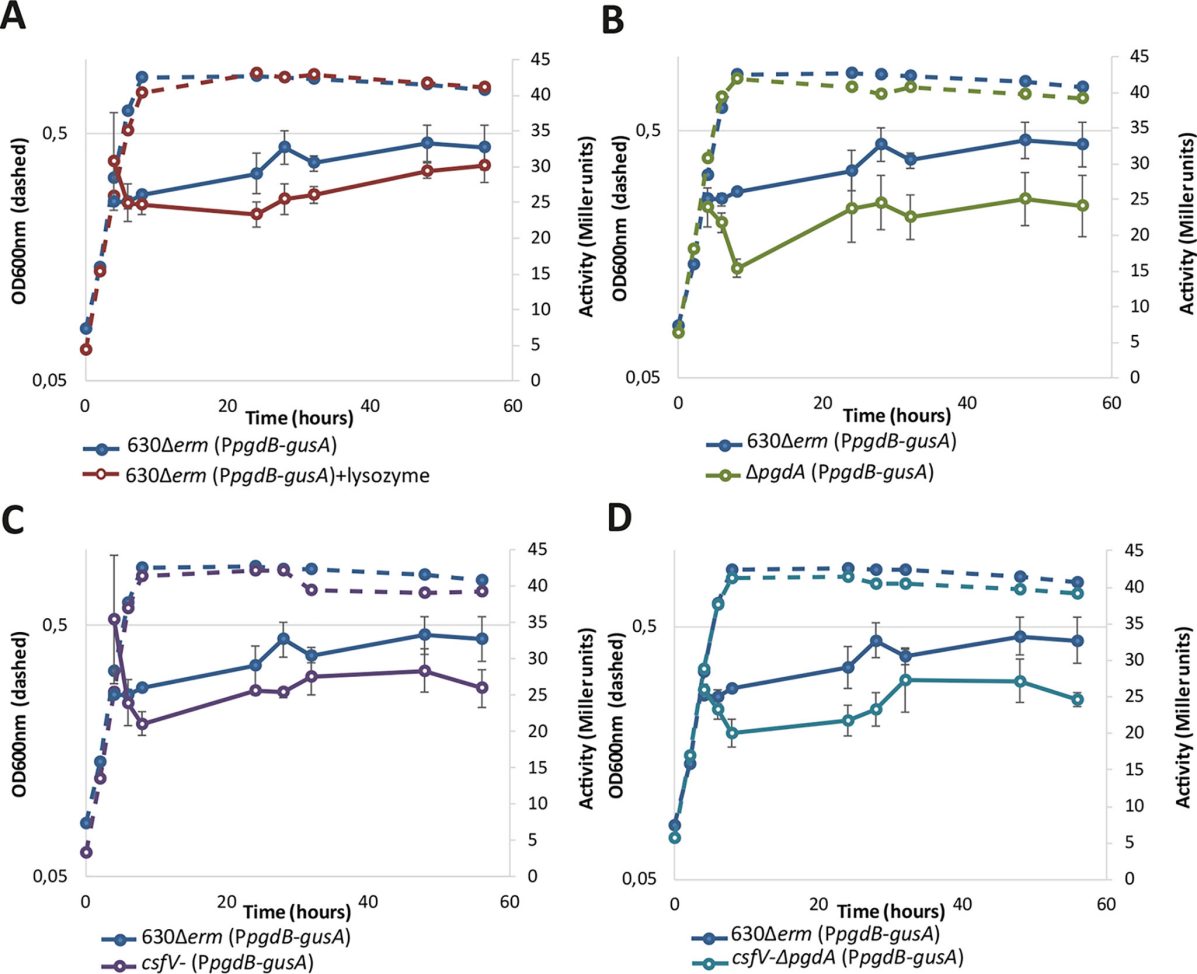
**β-Glucuronidase activity from P*pgdB*-*gusA* fusion.** We studied the β-glucuronidase activity from P*pgdB*-*gusA* fusion (pCH31) under four conditions: with lysozyme (*A*, *red*), in the Δ*pgdA* mutant (*B*, *green*), without σ^V^ (*C*, *purple*), and in the *csfV-*Δ*pgdA* double mutant (*D*, *cyan*), compared with the expression in the parental strain 630Δ*erm* (*dark blue*). The growth curve is shown in *dotted lines*, and the β-glucuronidase activity is shown in *solid lines*.

Because the genetic region of *pgdB* appears to have multiple potential promoters, we ran RT-PCR on three intergenic regions upstream of *pgdB*, and we used the intergenic region identified as promoter for the P*_pgdB_*-*gusA* plasmid (Fig. S3). In contrast with PgdA and PdaV, P*_pgdB_-gusA* activity was detected between 20 and 40 Miller units, which is only slightly over the detection threshold (Fig. 5*A*, *blue curve*). This result suggests that *pgdB* is very poorly expressed in these conditions. The P*_pgdB_*-*gusA* activity remained between 20 and 40 Miller units when lysozyme was added to the medium (Fig. 5*A*, *red curve*), which suggests that *pgdB* is not induced by lysozyme.

The *pgdB* expression did not show any significant variation in the Δ*pgdA* mutant, the *csfV-* mutant, or the *csfV-*Δ*pgdA* double mutant compared with its expression in the parental strain (Fig. 5, *A–D*). These results suggest that *pgdB* expression does not share the regulation patterns found for PgdA and PdaV and therefore that PgdB is not redundant with PgdA or/and PdaV. In addition, its transcription has been shown to be positively regulated by the sporulation sigma factor σ^E^ (26). We therefore investigated whether PgdB is able to deacetylate the GlcNAc from the cortex and found that the Δ*pgdB* mutant has decreased glucosamine deacetylation compared with the spore cortex from the parental strain (55.5% and 61.1%, respectively). However, because of high inter-replicate variability, the average *N*-deacetylation differences between both strains do not reach statistical significance. Altogether, these results suggested that PgdB could act as an GlcNAc deacetylase, but its target remains difficult to pinpoint.

### The csfV-ΔpgdA and csfV-ΔpgdAΔpgdB mutants have an increased virulence

**Figure 6:**
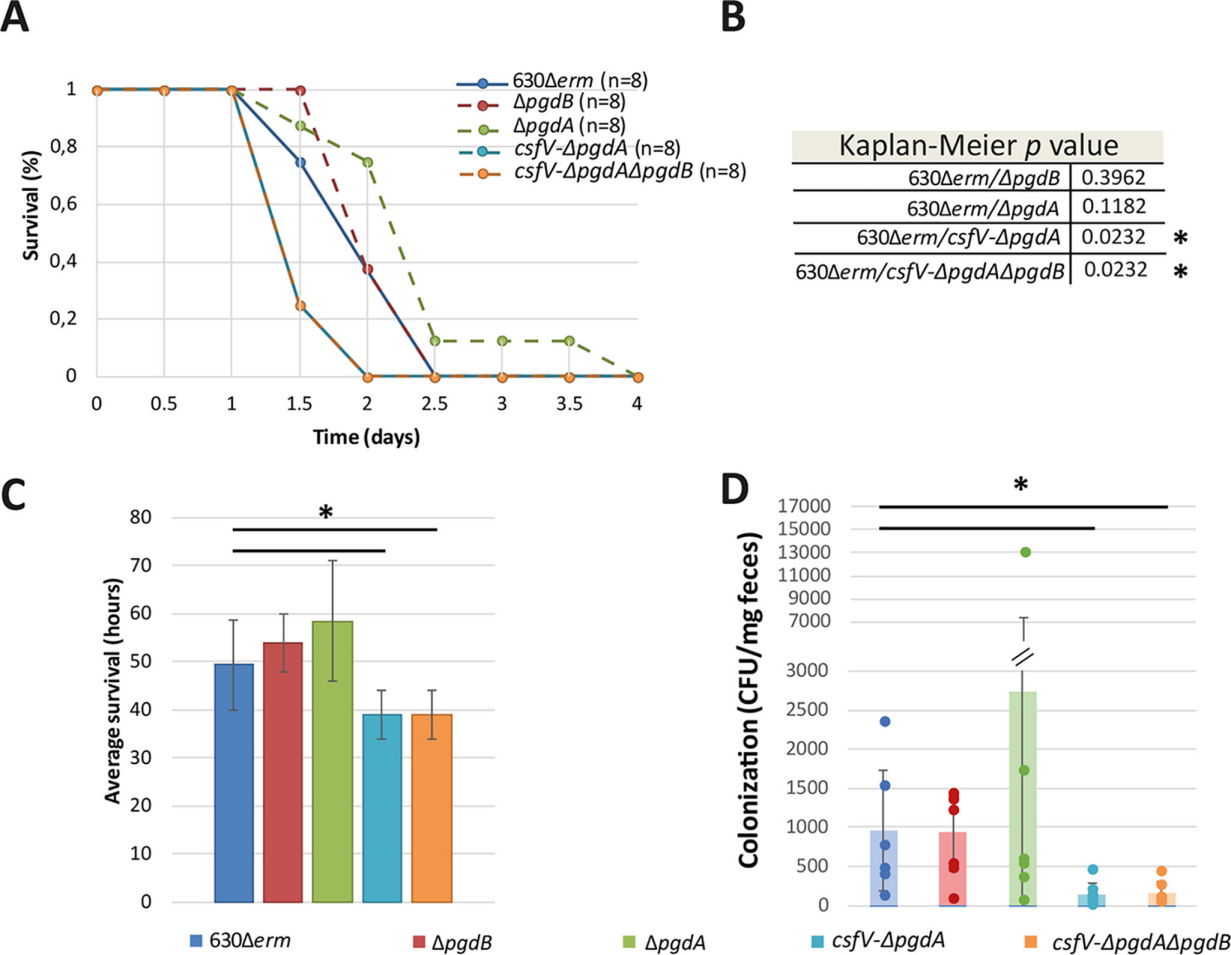
**The double and triple mutants have an increased virulence with a decrease in colonization.** The mutants are represented in *red* for Δ*pgdB*, in green for Δ*pgdA*, in *purple* for *csfV-*Δ*pgdA*, in *orange* for *csfV-*Δ*pgdA*Δ*pgdB*, and in *dark blue* for the parental strain 630Δ*erm*. *A*, Kaplan–Meier survival curve showing hamster survival over time for the parental strain in *solid line* and mutants in *dotted lines*. *Lines* corresponding to *csfV-*Δ*pgdA* and *csfV-*Δ*pgdA*Δ*pgdB* are superposed. *B*, Kaplan–Meier *p* values. *C*, average time to mortality. *D*, colonization assessment was obtained by serial dilution of fecal pellets plated on BHI agar supplemented with 25% (w/v) d-cycloserine, 0.8% (w/v) cefoxitin, and 1% defibrinated horse blood, and expressed as CFU/mg of feces. The results are represented as the means and standard deviations of the plating results for each hamster (*columns*) as well as the individual results obtained for the colonization level of each hamster (*scatter plot*). *, *p* < 0.05.

Because the mutants harbored different rates of *N*-deacetylated peptidoglycan, we investigated the contribution of Δ*pgdA* and Δ*pgdB*, as well as the *csfV-*Δ*pgdA* and *csfV-*Δ*pgdA*Δ*pgdB* mutants in pathogenesis in a hamster model of virulence (Fig. 6). After infection, all hamsters were tested for the presence of *C. difficile* through the on/off method, which allowed us to confirm carriage of *C. difficile* for every group of hamsters (5).

In our assay, mortality of the hamsters infected with the parental strain spores started at day 1 and lasted until day 2.5 (Fig. 6*A*). Although hamsters who received the Δ*pgdB* mutant spores showed a similar profile, the Δ*pgdA* group showed a slight reduction in mortality rate, without reaching statistically significant values (Kaplan–Meier log rank *p* = 0.11). In comparison, the double and triple mutant groups showed an earlier mortality by 1.5 days postinfection, with only 25% survival of hamsters in both groups compared with 75% survival of hamsters in the parental group. At 2 days postinfection, no survival was observed in the double and triple mutant groups, compared with 40% survival in the parental strain group. These results suggest that the double and triple mutant–infected hamsters reach mortality significantly faster than those who received the parental strain (Fig. 6*B*, 49.5 h ± 9.4 h for 630Δ*erm versus* 39.0 h ± 5.2 h for *csfV-*Δ*pgdA* and *csfV-*Δ*pgdA*Δ*pgdB*, Student's *t* test, *p* < 0.05). Surprisingly, colonization monitoring by plating serial dilution of fecal pellets revealed a lower CFU count in the fecal contents plated for the double and triple mutant strain, with averages of 143.0 ± 133 and 143.8 ± 135.8 CFU/mg of feces respectively, compared with 955.9 ± 767.6 CFU/mg of feces for the parental strain (Fig. 6*D*). Taken together, these results suggest that the *csfV-*Δ*pgdA* and *csfV-*Δ*pgdA*Δ*pgdB* mutants have an increased virulence (Fig. 6*A*, Kaplan–Meier log rank *p* = 0.0232, both groups), even though they show a decrease in colonization.

## Discussion

In this study, we reported that PdaV and PdgA are the main peptidoglycan *N*-deacetylases of *C. difficile*. The *pdaV* gene belongs to the *csfV* operon that also encodes σ^V^ and RsiV (21). RsiV is an anti-σ factor that inhibits σ^V^: when RsiV binds lysozyme, it releases σ^V^, which in turn activates the transcription of the σ^V^ operon, including its own transcription and *pdaV*. According to our results, there is a constitutive expression of this operon without lysozyme, suggesting that PdaV is produced and able to *N*-deacetylate the peptidoglycan. However, in the absence of lysozyme, the Δ*pgdA* single mutant was the one to show the strongest decrease in peptidoglycan *N*-deacetylation, suggesting that it is the major *N*-deacetylase of *C. difficile* in these conditions.

According to the lysozyme MIC of the *csfV-* and Δ*pgdA* simple mutants, PgdA and PdaV are both involved in lysozyme resistance. Because 70% of the peptidoglycan is still *N*-deacetylated in the Δ*pgdA* mutant, and *N*-deacetylation reaches 7.4% in the *csfV-*Δ*pgdA* double mutant, altogether our results indicated that there is a synergy of activity between both enzymes with and without the presence of lysozyme.

Although PdaV and PgdA appear to be *bona fide* peptidoglycan *N*-deacetylases, the role of PgdB is not as clear. The Δ*pgdB* mutant has not shown any phenotype in the conditions tested, and we did not see significant differences in peptidoglycan *N*-deacetylation between the double and triple mutant. Our results obtained for *pgdB* expression indicate that the *N*-deacetylase is not regulated in a pattern similar to what we observed for PgdA and PdaV. We therefore suggest that PgdB does not act as a third enzyme redundant with PgdA and PdaV.

However, expression of *pgdB* in the Δ*pgdA* mutant rescues the lysozyme sensitivity phenotype (Fig. 2*B*) without resulting in a detectable increase in peptidoglycan *N*-deacetylation (83.9% compared with 82.9% with the empty plasmid; Table 1). According to previously published data, *pgdB* is highly expressed *in vivo* after 14 and 38 h postinfection (27), and *pgdB* was also reported to be regulated by the sporulation factor σ*^E^* (26), and the corresponding protein is not detected in the free spore (28). We showed that the spore cortex of the Δ*pgdB* mutant was slightly decreased, but did not reach statistical significance, in GlcNAc deacetylation compared with the parental strain. A potential role of PgdB as a peptidoglycan *N*-deacetylase, expressed by the mother cell during the early phases of sporulation acting by deacetylation of the GlcNAc residues of the spore cortex cannot be excluded. This hypothesis would explain the ability of PgdB to act on lysozyme resistance.

Several *N*-deacetylases have been described as essential to the virulence of various pathogens, including PgdA in *S. pneumoniae* and *L. monocytogenes* (9, 29). For these pathogens, lack of *N*-deacetylation induces higher bacterial clearance and a strong decrease in virulence. In *C. difficile*, the *csfV-* mutant's ability to cause illness in a hamster model has already been assessed by two studies: although Ho *et al.* (21) reported decreased virulence, Woods *et al.* (25) reported increased virulence compared with the parental strain, with increased colonization. This conflicting result might be explained by a number of factors, including the different strain background used in each study. However, the *csfV-* mutant remained highly *N*-deacetylated in both our study and the peptidoglycan analysis published by Ho *et al.* (21). In our study, the relationship does not seem as straightforward: inactivating *csfV* or deleting *pgdA* does not result in significant differences in virulence. This could be explained by the high level of peptidoglycan *N*-deacetylation remaining for these mutants (92 and 73.7%, respectively).

In contrast, the double and triple mutants surprisingly showed increased virulence and decreased colonization *in vivo* even though they have a strongly reduced *N*-deacetylation level (7.4 and 4.6% of *N*-deacetylation, respectively). Our main hypothesis for this unexpected outcome is that the double and triple mutants have an increased *in vivo* bacterial lysis because of their lysozyme sensitivity, consistent with the decreased colonization. However, instead of leading toward bacterial clearance and host survival, the increased lysis could be associated with a strong inflammatory response as a consequence of massive pathogen-associated molecular pattern (PAMP) release. Indeed, previous studies in *L. monocytogenes* have shown that a lack of *N*-deacetylation increases recognition of peptidoglycan by NODs and a stronger downstream inflammatory host response (9, 10). Thus, the *in vivo* clearance of the double and triple mutants may release muropeptides that contribute to a highly inflammatory host response, which in turn may increase the symptoms leading to the swift death or euthanasia of the hamsters. The morphology defect seen for the double and triple mutants with lysozyme should also be taken into consideration in this hypothesis. Indeed, in mammals, lysozyme at mucosal surfaces can reach concentrations as high as 1 mg/ml (1). At this concentration, we have shown *in vitro* that the double and triple mutants are either cleared or form L-form like structures that are extremely fragile. Even though this is highly speculative, this phenomenon could very well occur *in vivo* as well and contribute to the release of inflammatory muropeptides. Consistent with this hypothesis, a strong inflammatory response has also been linked with increased intestinal epithelium injury as well as increased levels of inflammation biomarkers in the serum and ultimately an increase in CDI severity in human infections (30). Finally, it might be argued that the difference in virulence could also be linked to a difference in toxin production. However, in the study published by Woods *et al.* (25), the *csfV-* mutant also showed an increase in virulence, and the authors did not detect a difference in toxin production.

Collectively, our results shed light on the unexpected impact of peptidoglycan *N*-deacetylation for *C. difficile*. This is an important finding in the understanding of *C. difficile* infection pathophysiology: although peptidoglycan *N*-deacetylation has been considered as a therapeutic target in pathogenic bacteria such as *S. pneumoniae*, we provide evidence that this approach might not be appropriate for the treatment of *C. difficile* infection. Nonetheless, future research could examine the inflammatory response linked to the lack of peptidoglycan *N*-deacetylation in *C. difficile* to gain fundamental insights in the immune response during infection.

## Experimental procedures

### Bacterial strains and plasmids

The plasmids and strains used in this study can be found in Table S3. The *C. difficile* strains are all isogenic derivatives of the 630Δ*erm* strain (31), an erythromycin-sensitive derivative of the clinical 630 strain (32). *E. coli* was grown aerobically at 37 °C in LB medium, supplemented with ampicillin (100 μg/ml), kanamycin (40 μg/ml), and chloramphenicol (25 μg/ml) as needed. *C. difficile* was routinely grown in brain–heart infusion (BHI) medium (BD), BHI supplemented with yeast extract, l-cysteine, and glucose (BHISG, as described in Ref. 24; or sporulation medium, as described in Ref. 33; or sporulation medium C, as described in Refs. 34 and 35). Mutant selection steps of the allelic exchange mutagenesis were carried out on *C. difficile* minimal medium (36) supplemented with 5-fluoro-cytosine (50 μg/ml). The media were supplemented with thiamphenicol (15 μg/ml), 0.1% sodium taurocholate (Sigma–Aldrich), 1% defibrinated horse blood, or *C. difficile* selective supplement (25% [w/v] d-cycloserine, 0.8% [w/v] cefoxitin; OXOID) when required. Cultures of *C. difficile* were carried out at 37 °C in an anaerobic chamber (Jacomex, 5% H2, 5% CO_2_, 90% N2).

### Molecular biology

The plasmid constructions are detailed in Supporting Data File S1.

### β-Glucuronidase assay

The GUS reporter system was used to analyze the activity of gene promoters. The method is based on the spectrophotometric detection of β-glucuronidase activity: incubation of the GusA protein with the colorless substrate *p*-nitrophenyl β-d-glucuronide leads to production of a yellow product. *C. difficile* strains containing the promoter-reporter *gusA* gene fusions listed in Table S3 were grown for 56 h. When mentioned, lysozyme was added in the growth medium from the start of the culture. As needed, 0.5–1 ml samples were harvested, and pelleted cells were stored at −20 ˚C. The samples were analyzed for β-glucuronidase activity following the described protocols (37, 38). The β-glucuronidase activity is expressed in Miller units. The results are presented as the means and standard deviations of three biological replicates.

### Lysozyme growth curves

Lysozyme sensitivity was assessed through growth curves using BHISG liquid medium supplemented with 1 mg/ml lysozyme. For the standard lysozyme growth assay, overnight strains were diluted in BHISG medium to obtain *A*_600nm_ = 0.05, lysozyme was added to obtain a final concentration of 1 mg/ml, and *A*_600nm_ was monitored for 8 h. In the complementation lysozyme assays, strains containing the appropriate plasmids were grown in BHISG overnight supplemented with thiamphenicol 5. In the morning, they were then diluted in BHISG medium and thiamphenicol 5 to obtain *A*_600nm_ = 0.05. For each strain, cultures were prepared in duplicate, and anhydrous tetracycline was added at a final concentration of 100 ng/ml in the “induced” cultures. After 3 h of growth, each culture was divided in two separate tubes, and lysozyme was added at the final concentration of 1 mg/ml in one of the divided tubes. Optical density was then monitored for an additional 5 h. The results are expressed as the means and standard deviations of three biological replicates.

### MIC assays

To determine MIC for lysozyme, the strains were grown overnight in BHISG liquid medium. The cultures were then diluted in medium to obtain *A*_600nm_ = 0.05, and different concentrations of lysozyme were added in 96-well plates. Positive wells were considered as a visual growth after 20 h of incubation. MIC of a given condition was determined as the last well for which no growth was observed. The results are represented as the means and standard deviations of at least three biological replicates.

### Peptidoglycan extraction and analysis

The samples for the vegetative cell peptidoglycan extraction were prepared as follows: for each strain, overnight cultures in sporulation medium were diluted to obtain *A*_600nm_ = 0.05 and incubated at 37 °C. For the complemented strains, anhydrous tetracycline was added in the diluted culture at a final concentration of 100 ng/ml. After 6 h of growth, 500 ml of exponential cultures (*A*_600nm_ between 0.5 and 0.7) were centrifuged and stored at −20 °C. Peptidoglycan was extracted from bacterial pellets according to the protocol described by Candela and Fouet (39). Peptidoglycan was then digested for 16 h with mutanolysin in a sodium phosphate buffer. The suspensions were centrifuged, and muropeptides contained in the supernatant were freeze-dried for 16 h (Telstar cryodos). Soluble muropeptides were then mixed with an equal volume of 500 mm borate buffer (pH 9) and reduced with sodium borohydride (NaBH_4_). After 30 min at room temperature, the pH was adjusted to 3 using orthophosphoric acid (H_3_PO_4_). After centrifugation, reduced muropeptides were diluted 2-fold for the first batch and 10-fold for the batch of biological replicates in mobile phase (0.1% formic acid in water). Spore cortex extractions were conducted as described in Refs. 5 and 40. Muropeptides extracted for the vegetative cell and spore cortex were analyzed by LC–high-resolution mass spectrometry (Thermo QExactive focus), as described in Ref. 5.

### In vivo virulence assay

Spore preparations for the *in vivo* virulence study were obtained according to the protocol described by Coullon *et al.* (5), stored at 4 °C, and numerated prior to infection of the animals. Adult female Syrian golden hamsters were housed in sterile individual cages. The absence of *C. difficile* was monitored before starting the assay. To induce susceptibility to infection with *C. difficile*, the hamsters were treated with clindamycin (0.5 ml, 10 mg/kg) 5 days before infection by oral administration. For each animal group, eight hamsters were infected orally by administration of 1 × 10^4^ spores of either the parental or the mutant strains (Δ*pgdA*, Δ*pgdB*, *csfV-*, *csfV-*Δ*pgdA*, and *csfv-*Δ*pgdA*Δ*pgdB*). Enumeration of spore suspensions were conducted on solid medium, after 48 h of incubation at 37 °C. After infection, the hamsters were monitored at least twice a day. *C. difficile* presence was monitored using two methods. It was first assessed through an on/off test: fecal pellets from each hamster were cultured in BHI supplemented with 0.1% sodium taurocholate for 12 h and plated on BHI agar plates supplemented with 25% (w/v) d-cycloserine, 0.8% (w/v) cefoxitin, and 1% defibrinated horse blood. Typical fluorescent colonies were screened under UV light (312 nm). In the second method used, colonization was quantified by plating serial dilution of fresh fecal pellets when available. Fecal pellets were resuspended in PBS separately for each animal (20 mg/ml), 10-fold serial diluted, and plated onto BHI agar plated supplemented with 25% (w/v) d-cycloserine, 0.8% (w/v) cefoxitin, and 1% defibrinated horse blood. All of the plates were incubated for 48 h at 37 °C in the anaerobic chamber. By using this method, the threshold of *C. difficile* detection in fecal pellets was 500 CFU/g. However, on/off testing ensured detection of colonization regardless of the threshold.

### Ethics statement

Adult female Syrian golden hamsters (95–105 g, Charles River France) were housed in individual sterile cages in an animal biosafety level 2 facility within the central animal facility of the pharmacy faculty, according to European Union guidelines for the handling of laboratory animals (http://ec.europa.eu/environment/chemicals/lab_animals/home_en.htm), and procedures for infection, euthanasia, and specimen collection were approved by the Ethics Committee CAPSUD (Protocol APAFiS no. 7492-2016101014285698).

### Statistics

Statistical analyses, including the Kaplan–Meier survival analysis, were carried out using the online statistical analysis service BiostaTGV (RRID:SCR_019094). The *P* value is indicated for all comparisons when the differences were found to be statistically significant.

## Data availability

The full raw data of muropeptides detected in the peptidoglycan and cortex analyses of every replicate have been deposited in MassIVE under accession number MSV000086027. All other data are contained within the manuscript.
