# Supplementary material for: Peptidoglycan analysis reveals that synergistic deacetylase activity in vegetative Clostridium difficile impacts the host response
Source: J Biol Chem. 2021 Jan 13;295(49):16785–96. doi: 10.1074/jbc.RA119.012442 (PMC7864072; doi:10.1074/jbc.RA119.012442)
Supplement: Supplementary file 1 [file mmc1.zip › 157925_2_supp_601609_qh3rdf.pdf]

Supplementary data

1 Structural informations

1.1 Unusual Amino Acids in muropeptides

1.1.1 Isoleucine/Leucine

(GMTripeptide + I/L) deacetylated

$[M+2H]^{2+}$ = 471.7293; Neutral mass = 941.4441; RT= 19.05 min

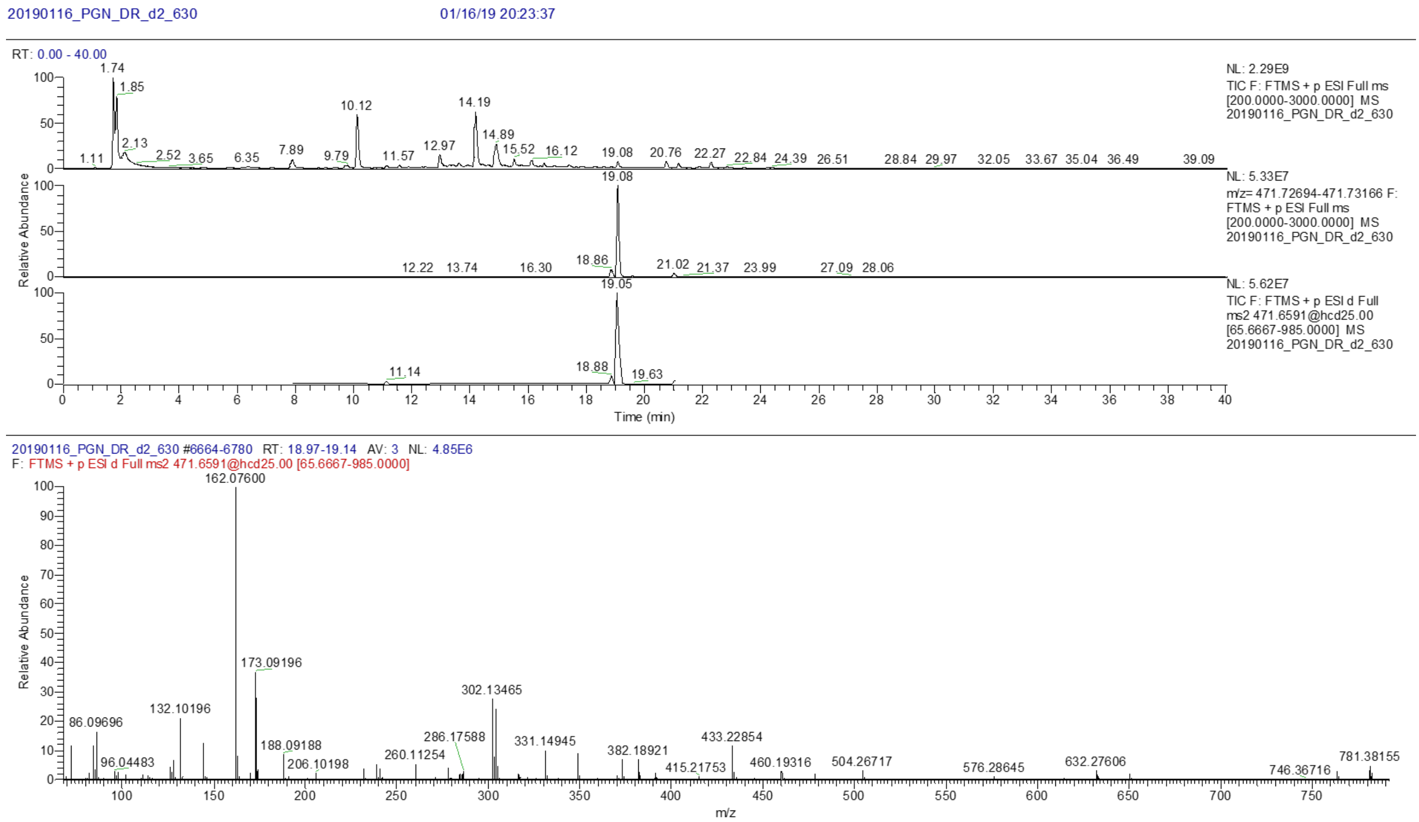

Figure 1: Sample#630; XIC of m/z 471.7293 and its MS/MS fragmentation spectrum; Normalized collision energy (NCE) = 25%

m/z obs. in MS/MS spectrum	residue	m/z th.	Error ppm
86.09696	I/L immonium ion	86.09698	-0.23
162.07600	Glucosamine + H^+	161.06881 + 1.00783 = 162.07664	-3.95
173.09196	A_2pm (mesoDap) + H^+	172.08479 + 1.00783 = 173.09262	-3.81
286.17588	(I/L)A_2pm + H^+	113.08406 + 172.08479 + 1.00783 =286.17668	-2.80
302.13465	A_2pmE + H^+	172.08479 + 129.04259 +1.00783 = 302.13521	-1.85
433.22854	(I/L)A_2pmE + H_2O + H^+	131.09463 + 172.08479 + 129.04259 + 1.00783 = 433.22984	-3.00

GMTripeptide + I/L

$[M+2H]^{2+}$ = 492.7346; Neutral mass = 983.4547; RT= 20.30 min

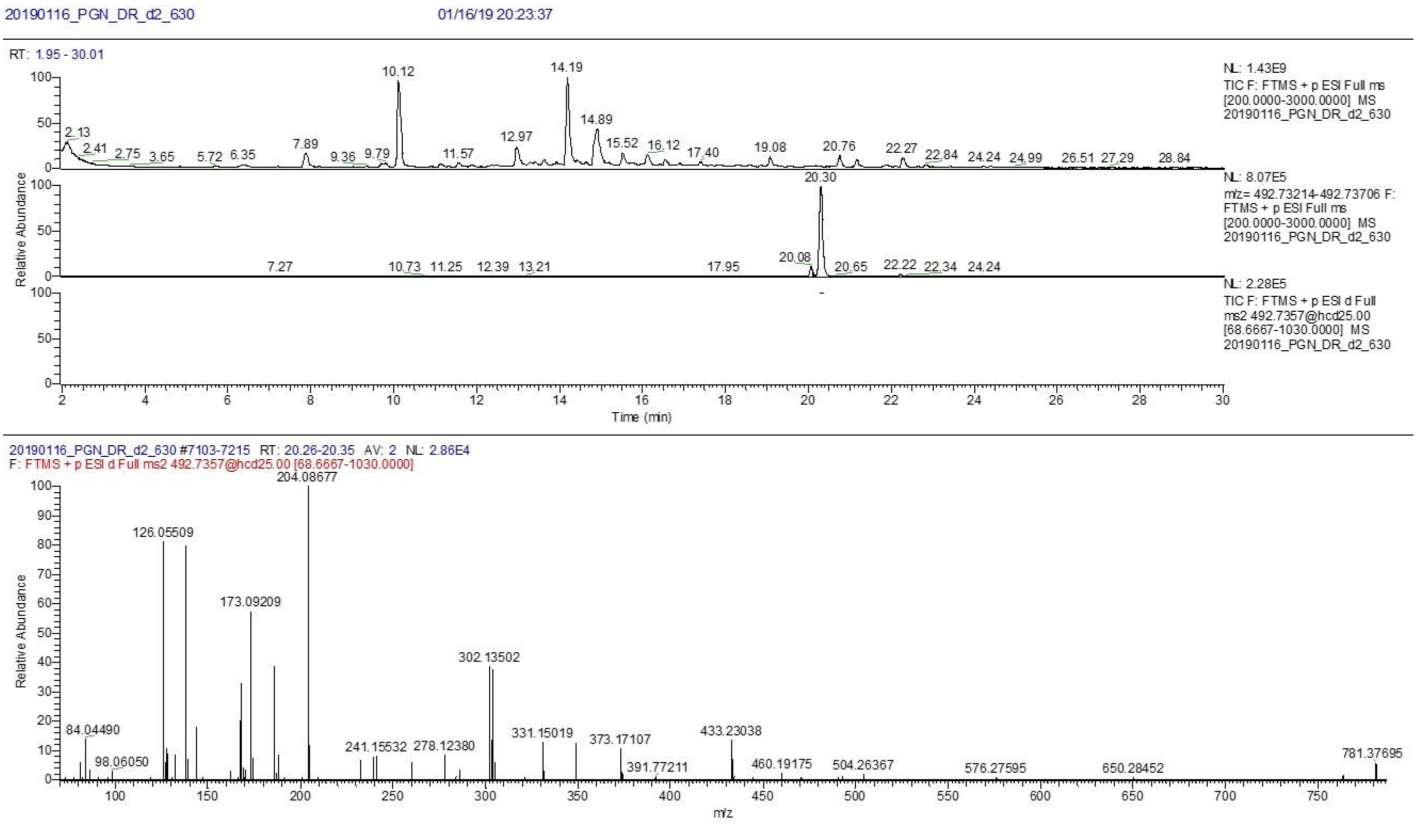

Figure 2: Sample#630; XIC of m/z 492.7346 and its MS/MS fragmentation spectrum; NCE 25%

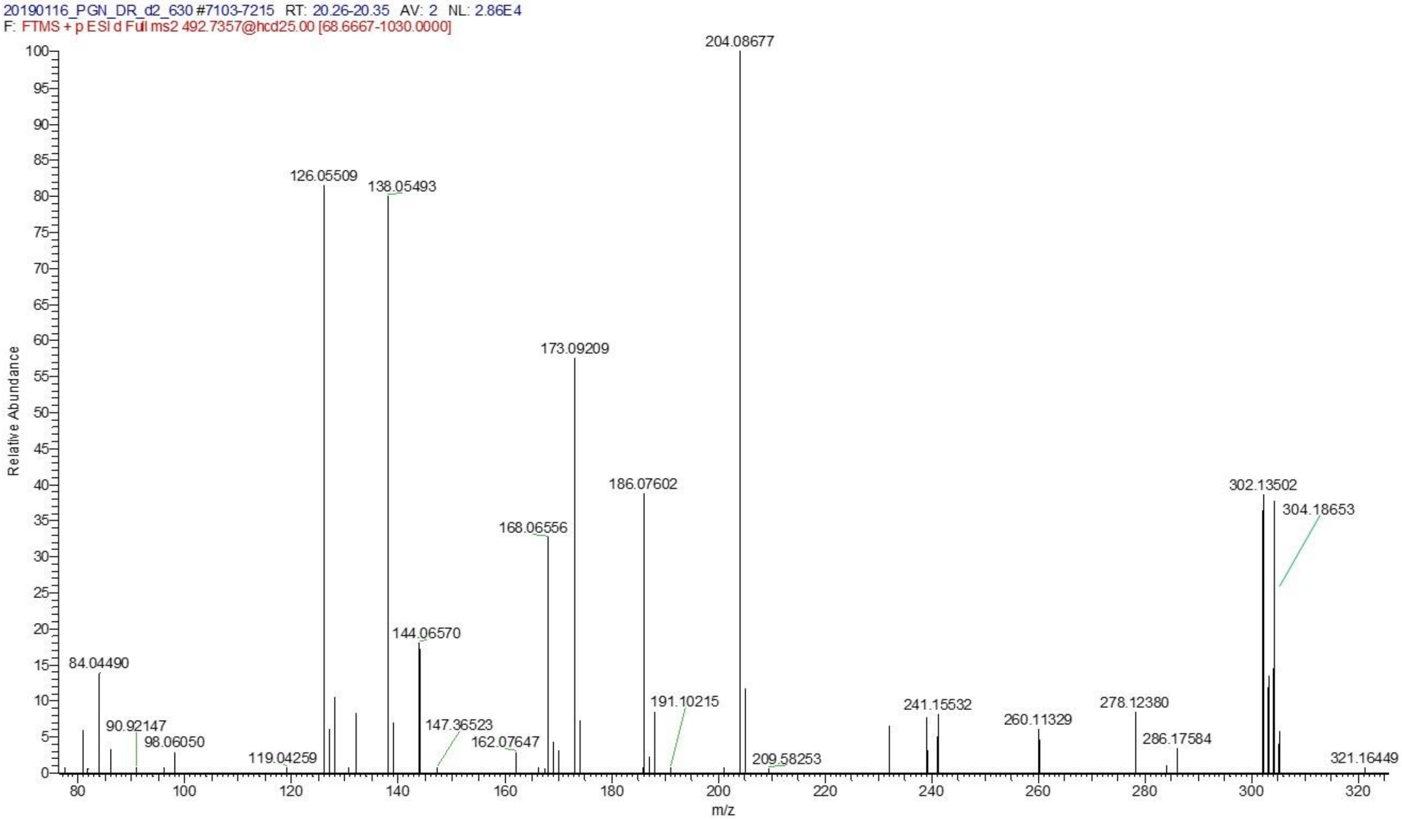

Figure 3: Zoom in low m/z

m/z obs. in MS/MS spectrum	residue	m/z th.	Error ppm
86.09687	I/L immonium ion	86.09698	-1.28
204.08677	GlcNAc + H^+	203.07937 + 1.00783 = 204.08720	-2.11

173.09209	A_2pm (mesoDap) + H^+	172.08479 + 1.00783 = 173.09262	-3.06
286.17588	(I/L)A_2pm + H^+	113.08406 + 172.08479 + 1.00783 =286.17668	-2.80
302.13502	A_2pmE + H^+	172.08479 + 129.04259 +1.00783 = 302.13521	-0.63
304.18653	(I/L)A_2pm + H_2O + H^+	131.09463 + 172.08479 + 1.00783 = 304.18725	-2.37
433.23038	(I/L)A_2pmE + H_2O + H^+	131.09463 + 172.08479 + 129.04259 + 1.00783 = 433.22984	1.25

(GMTripeptide + I/L) deacetylated

$[M+2H]^{2+}$ = 471.7292; Neutral mass = 941.4441; RT= 16.82 min; error ppm = -0.21

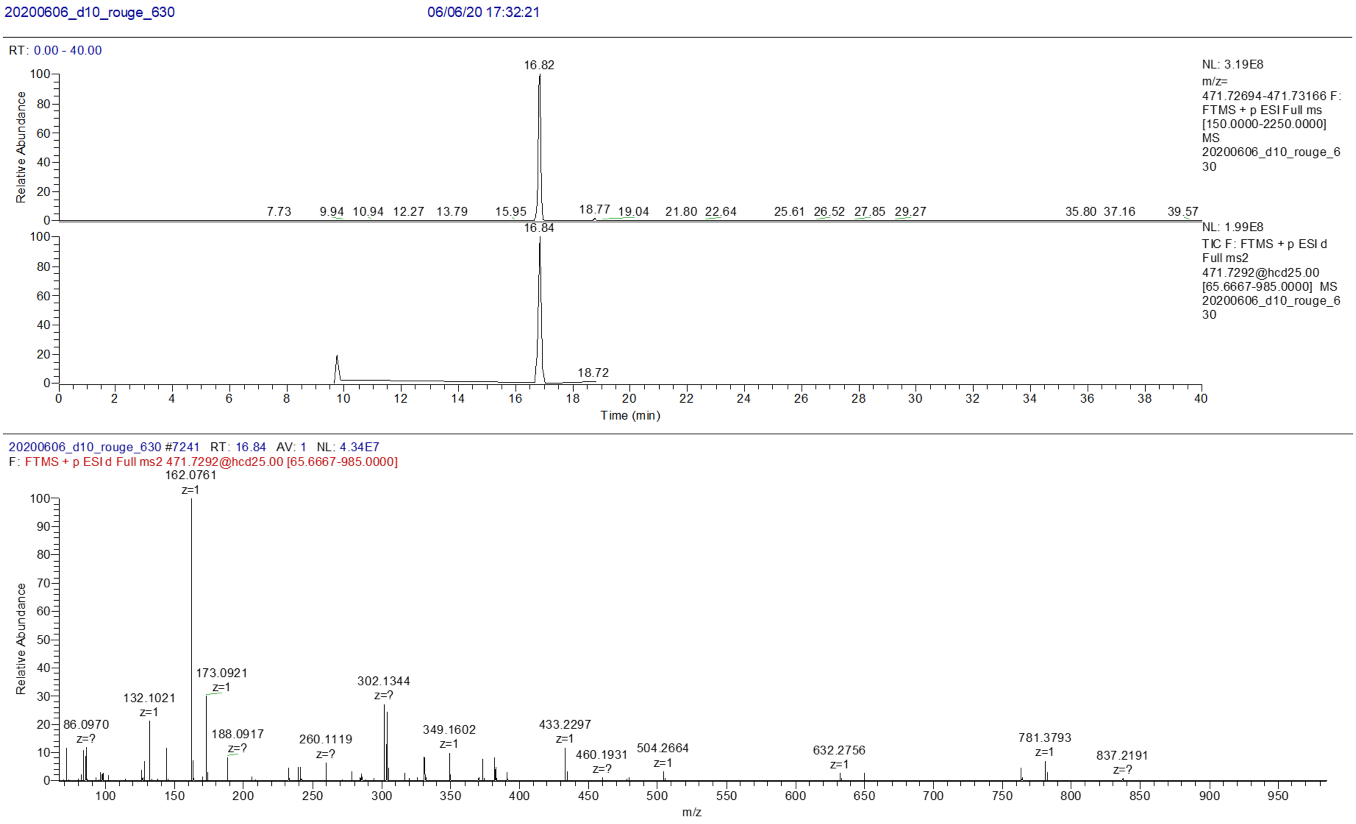

Figure 4: Sample#630; XIC of m/z 471.7293 and its MS/MS fragmentation spectrum. New analysis 2020_06.

GMTripeptide + I/L

$[M+2H]^{2+}$ =492.7348; Neutral mass = 983.4547; RT= 18.50 min; error ppm = 0.41

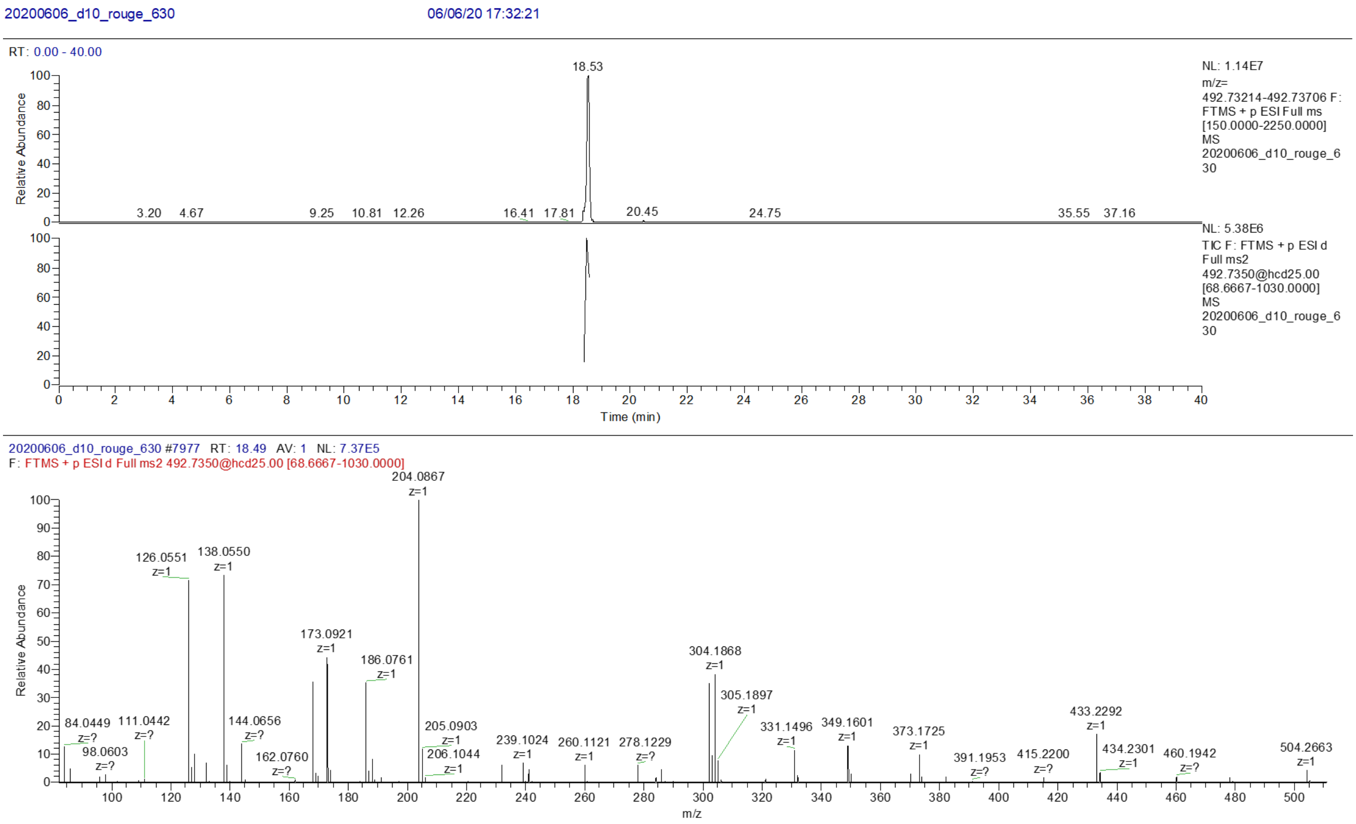

Figure 5: m/z 492.7350 and its MS/MS fragmentation spectrum. New analysis 2020_06

1.1.2 Phenyalanine

(GMTripeptide + F) deacetylated

$[M+2H]^{2+}$ = 488.7215; Neutral mass = 975.4284; RT= 20.75 min

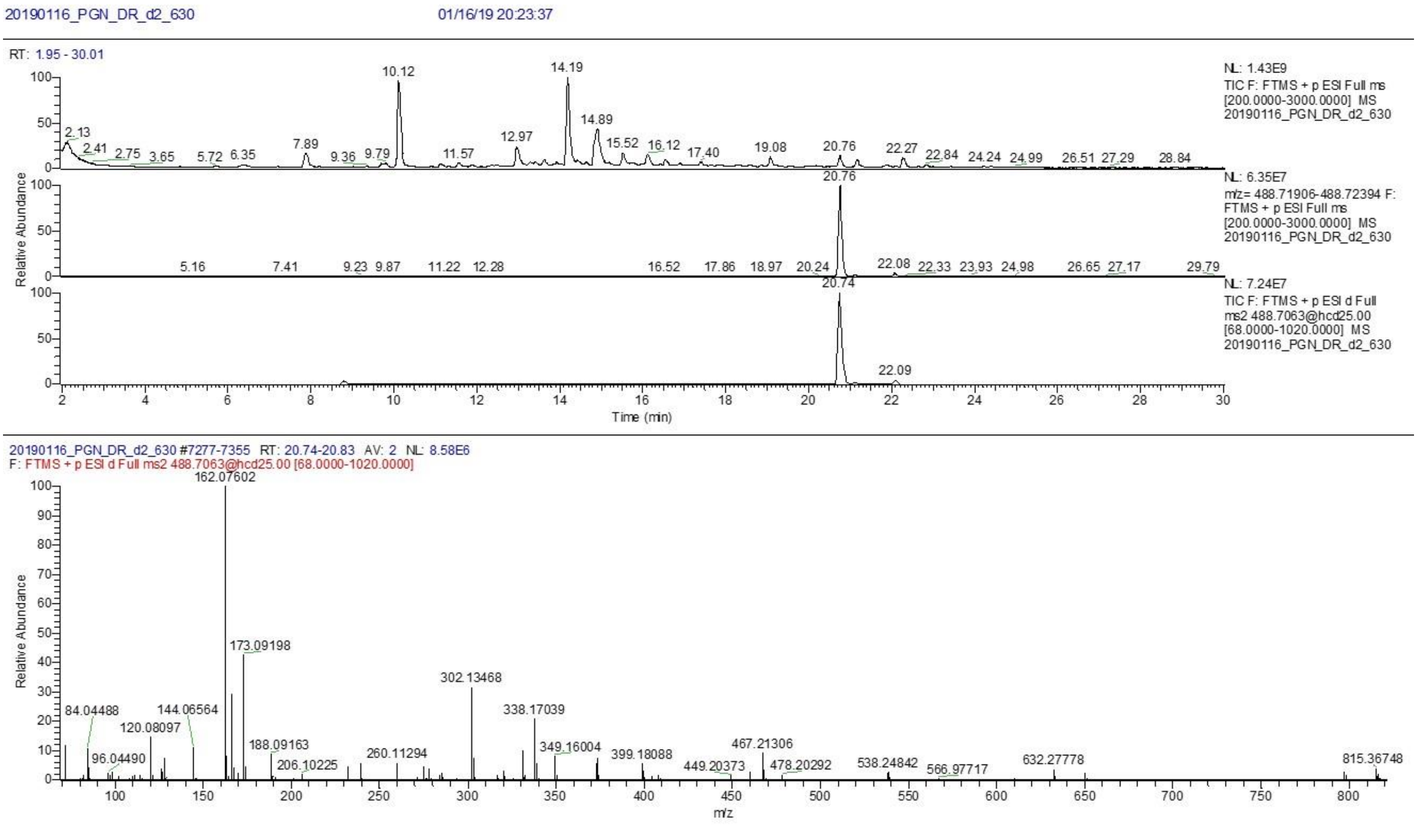

Figure 6: Sample#630; XIC of m/z 488.7215 and its MS/MS fragmentation spectrum; NCE 25%

m/z obs. in MS/MS spectrum	residue	m/z th.	Error ppm
120.08097	F immonium ion	120.08130	-2.75
162.07602	Glucosamine + H^+	161.06881 + 1.00783 = 162.07664	-3.83
173.09198	A_2pm (mesoDap) + H^+	172.08479 + 1.00783 = 173.09262	-3.70
302.13468	A_2pmE + H^+	172.08479 + 129.04259 + 1.00783 = 302.13521	-1.75
338.17039	FA_2pm + H_2O + H^+	165.07899 + 172.08479 + 1.00783 = 338.17160	-3.58
467.21306	FA_2pmE + H_2O + H^+	165.07899 + 172.08479 + 129.04259 + 1.00783 = 467.21419	-2.42

GMTripeptide + F

$[M+2H]^{2+}$ = 509.7268; Neutral mass = 1017.4390; RT= 22.10 min

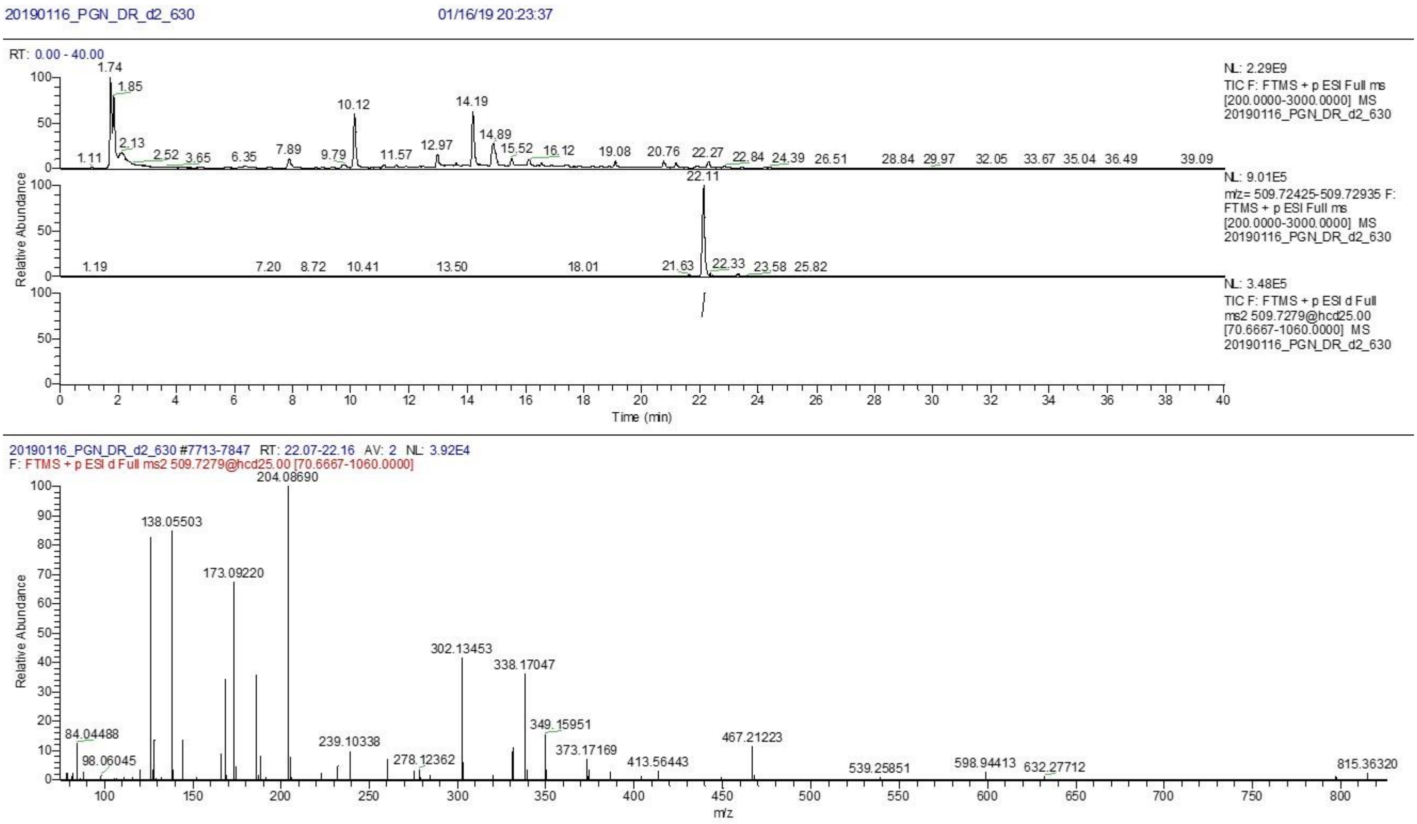

Figure 7: Sample#630; XIC of m/z 509.7268 and its MS/MS fragmentation spectrum; NCE 25%

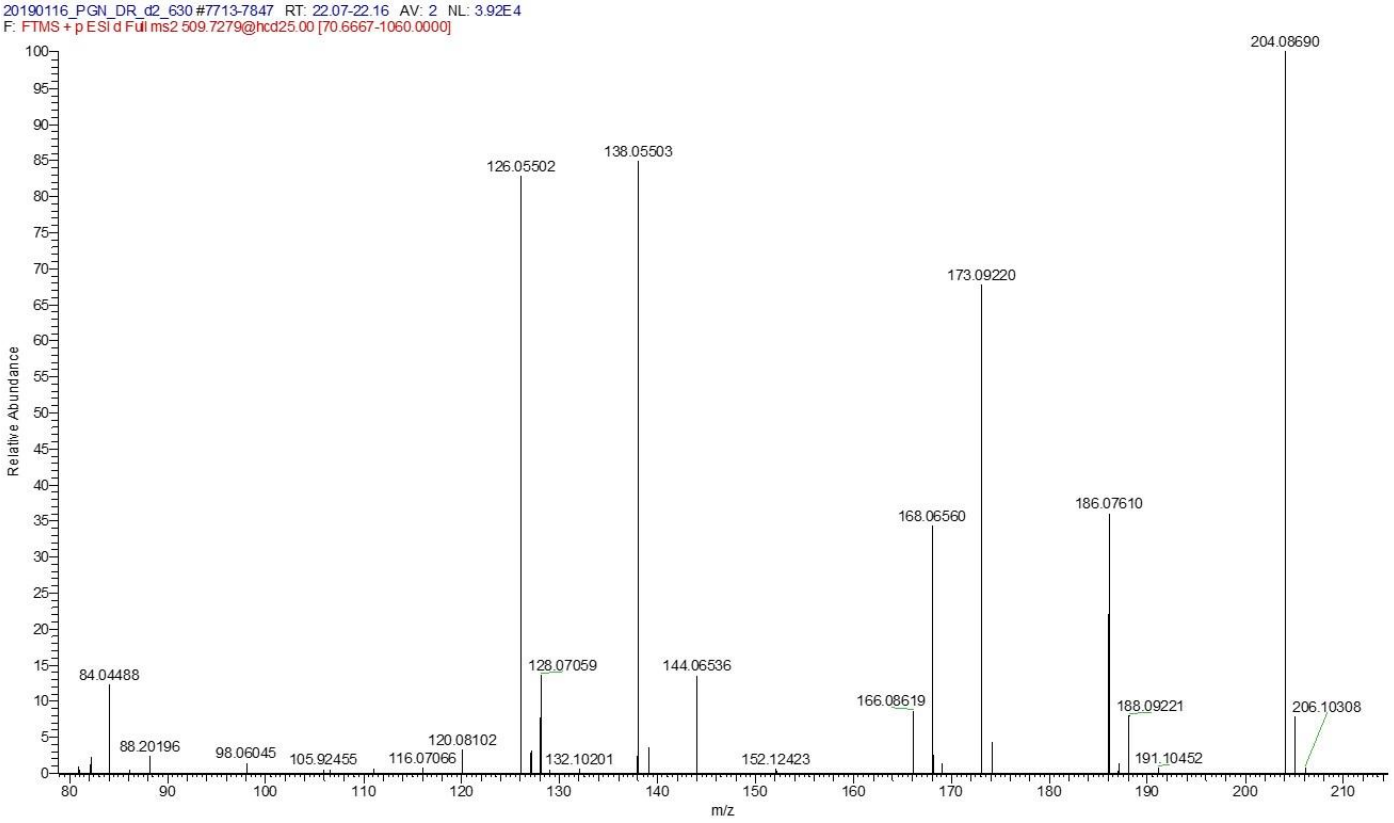

Figure 8: Zoom in the low m/z in MS/MS spectrum

m/z obs. in MS/MS spectrum	residue	m/z th.	Error ppm
120.08102	F immonium ion	120.08130	-2.33
173.09220	A_2pm (mesoDap) + H^+	172.08479 + 1.00783 = 173.09262	-2.43

204.08690	GlcNAc + H^+	203.07937 + 1.00783 = 204.08720	-1.47
302.13453	A_2pmE + H^+	172.08479 + 129.04259 + 1.00783 = 302.13521	-2.25
338.17047	FA_2pm + H_2O + H^+	165.07899 + 172.08479 + 1.00783 = 338.17160	-3.34
467.21223	FA_2pmE + H_2O + H^+	165.07899 + 172.08479 + 129.04259 + 1.00783 = 467.21419	-4.20

(GMTripeptide + F) deacetylated

$[M+2H]^{2+}$ = 488.7218; Neutral mass = 975.4284; RT= 18.75 min; error ppm = 0.61

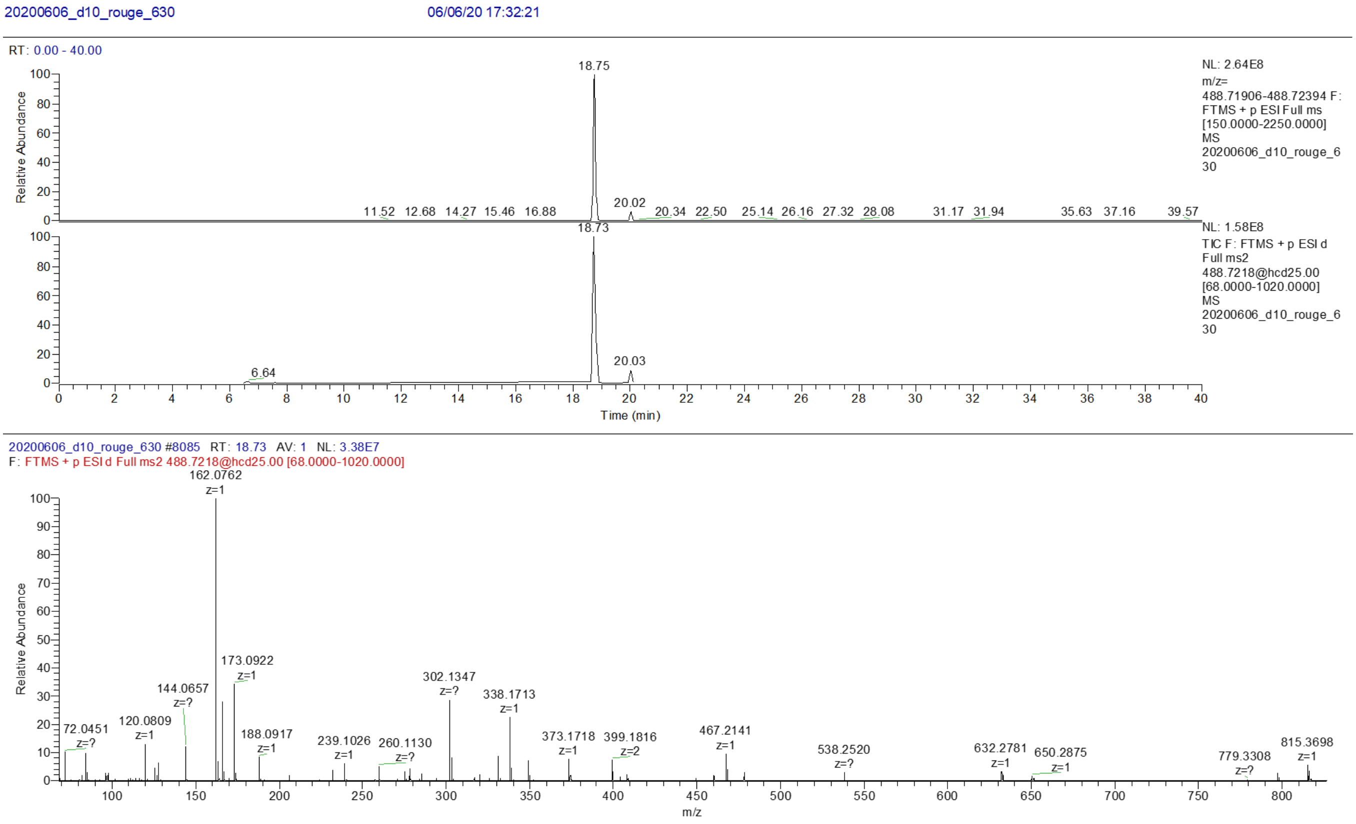

Figure 9: MS/MS fragmentation of m/z 488.7218

GMTripeptide + F

$[M+2H]^{2+}$ = 509.7268; Neutral mass = 1017.4390; RT= 20.62 min; error ppm =1.37

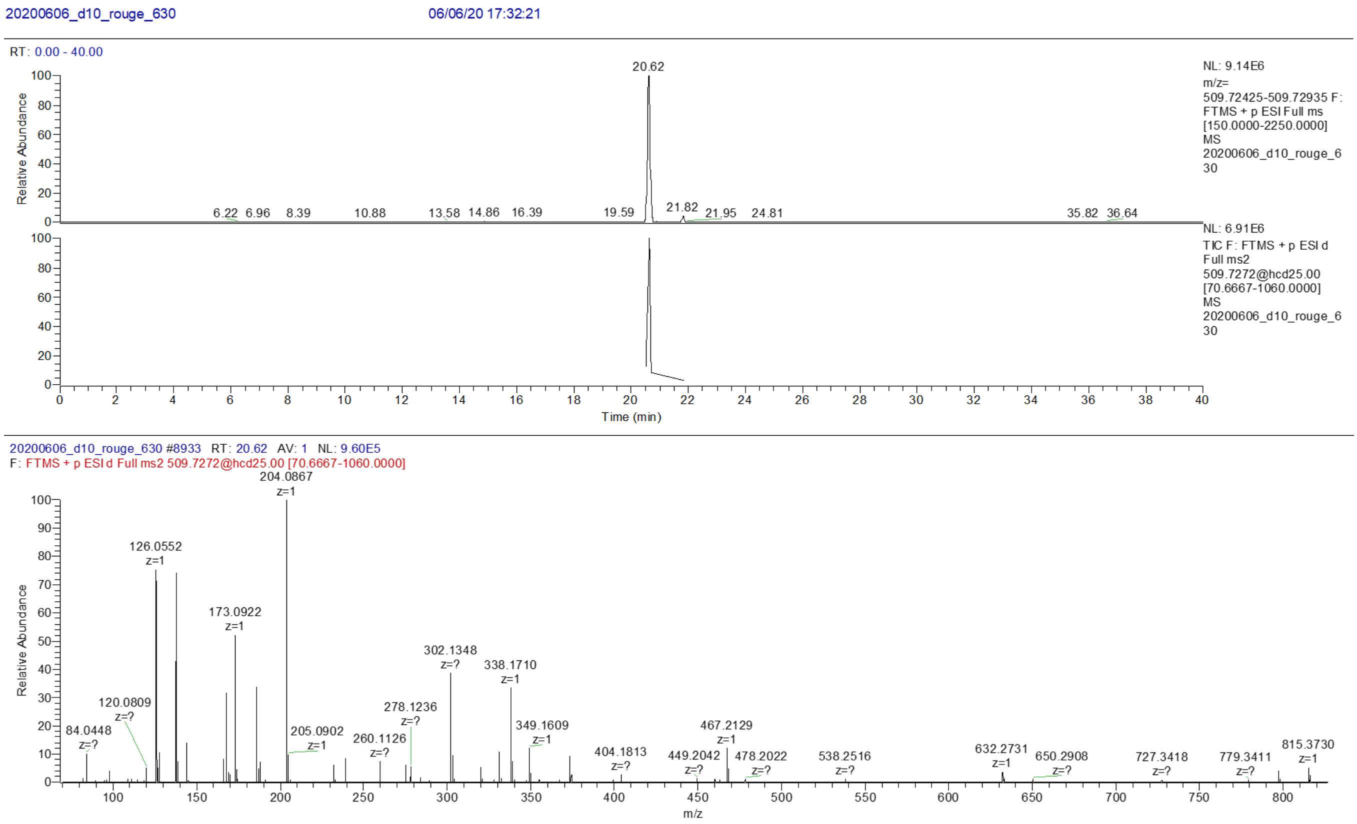

Figure 10 : New analysis 2020_06

1.1.3 Valine

(GMTripeptide + V) deacetylated

$[M+2H]^{2+}$ = 464.7215; Neutral mass = 927.4284; RT= 15.20 min

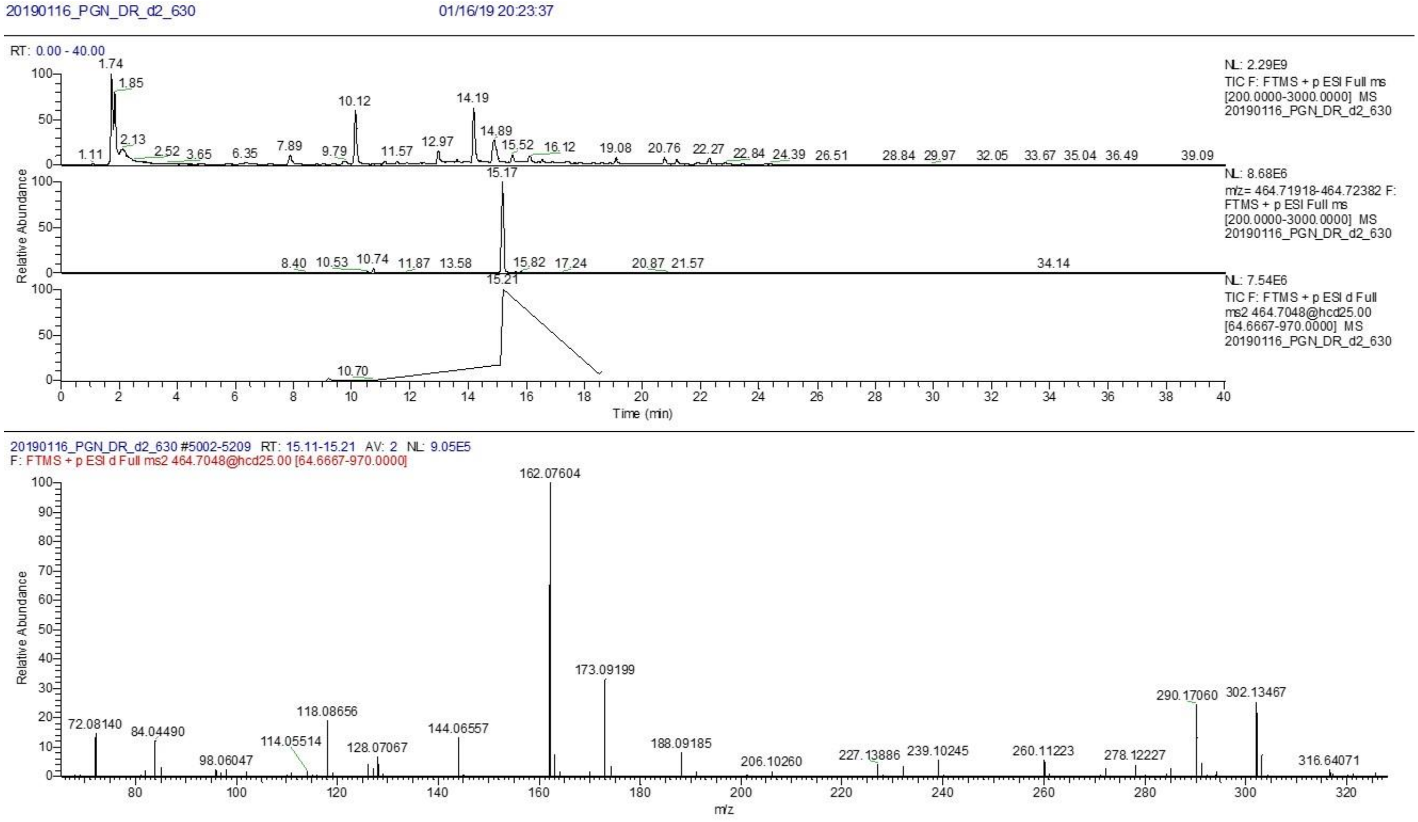

Figure 11: MS/MS fragmentation of GM3+Valine deacetylated.

m/z obs. in MS/MS spectrum	residue	m/z th.	Error ppm
72.08140	V immonium ion	72.08133	0.97
118.08656	V + H_2O + H^+	99.06871 + 18.01057 + 1.00783 = 118.08681	-4.23
162.07604	Glucosamine + H^+	161.06881 + 1.00783 = 162.07664	-3.70
173.09199	A_2pm (mesoDap) + H^+	172.08479 + 1.00783 = 173.09262	-3.64
290.17060	VA_2pm + H_2O + H^+	117.07898 + 172.08479 + 1.00783 = 290.17160	-3.45
302.13467	A_2pmE + H^+	172.08479 + 129.04259 + 1.00783 = 302.13521	-1.79

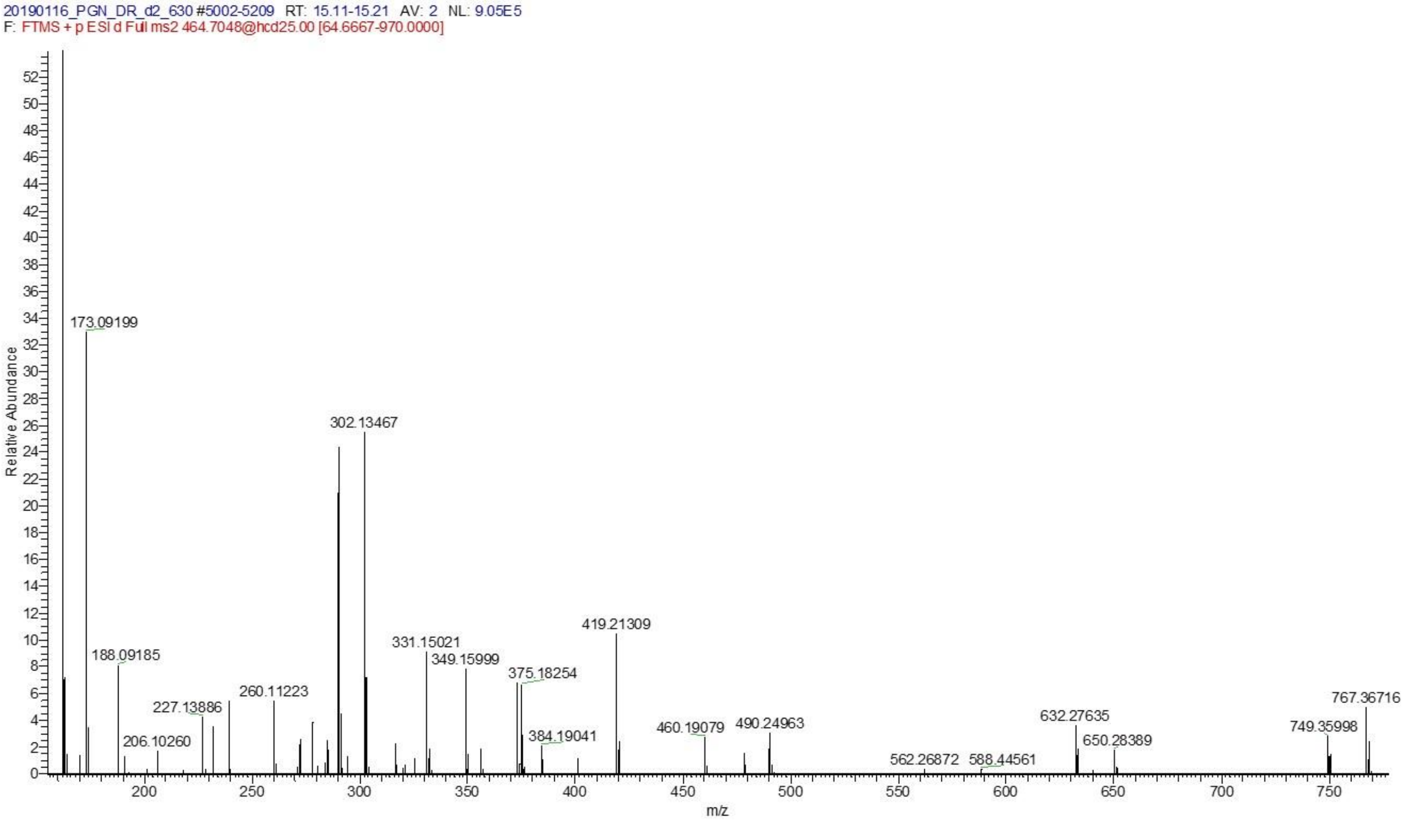

Figure 12: zoom in MS/MS spectra.

GMTripeptide + V

$[M+2H]^{2+}$ = 485.7268; Neutral mass = 969.4390; RT= 16.25 min

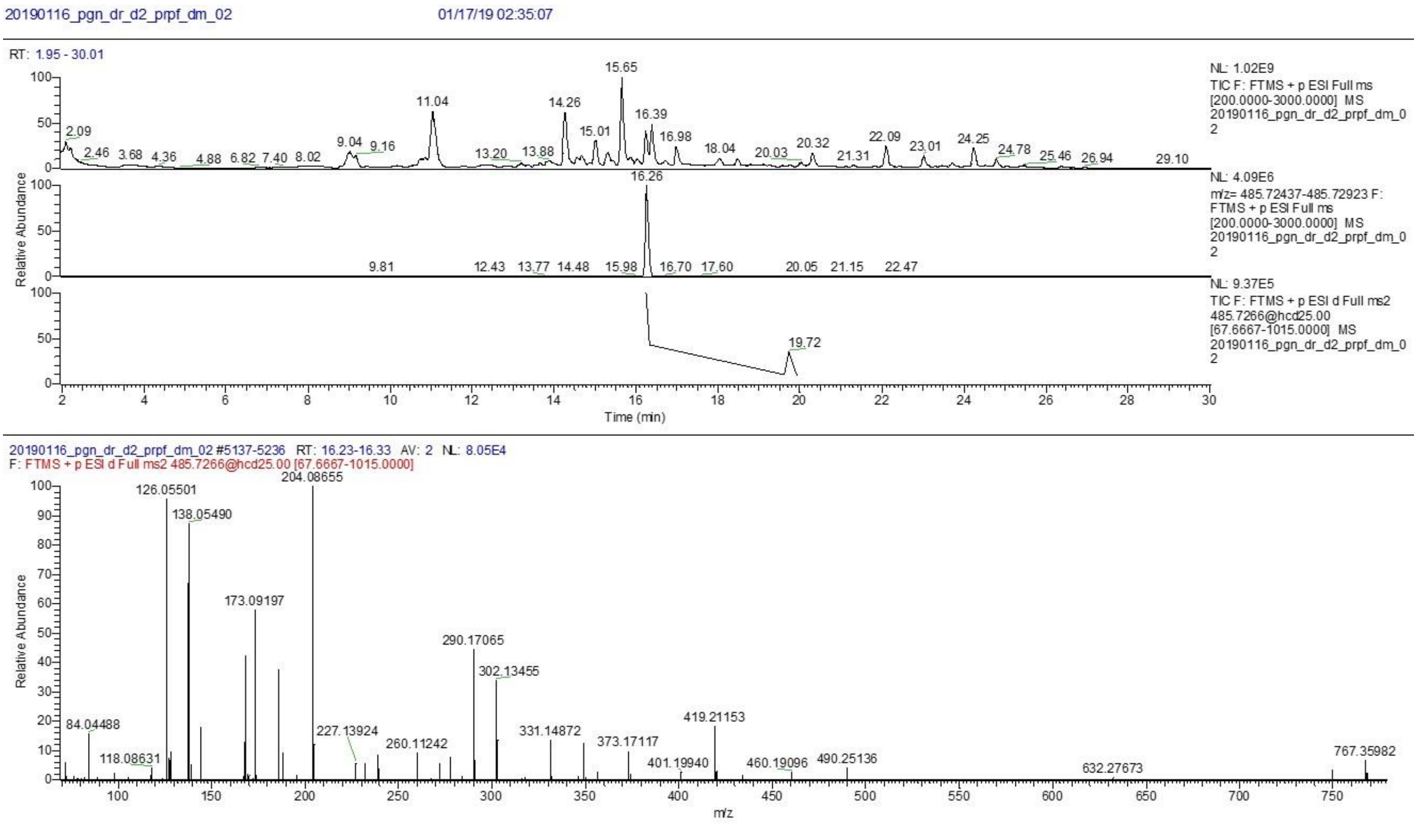

Figure 13: MS/MS of its non deacetylated counterpart.

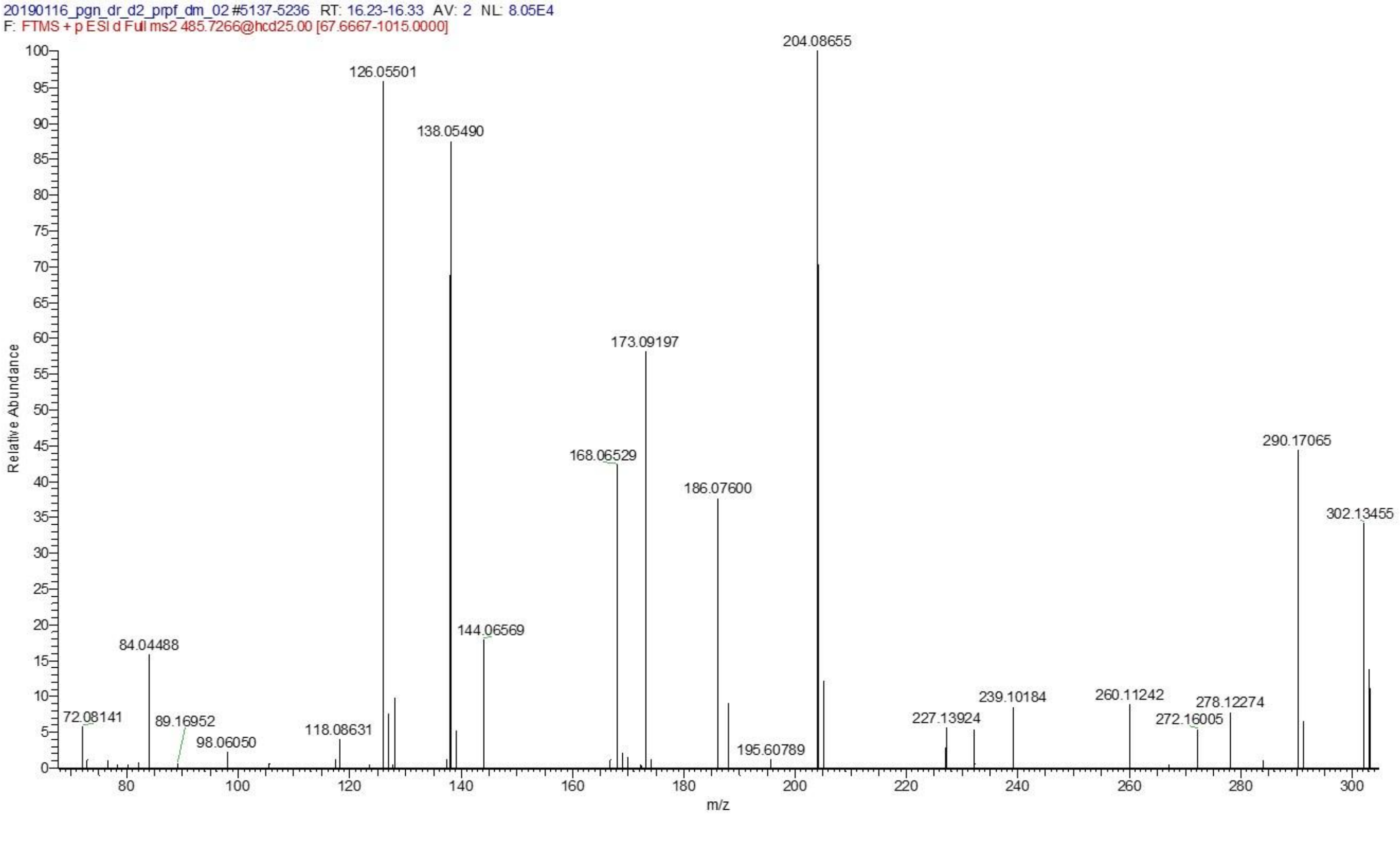

Figure 14: zoom in low m/z range of MS/MS spectra.

m/z obs. in MS/MS spectrum	residue	m/z th.	Error ppm
72.08141	V immonium ion	72.08133	1.11
118.08631	V + H_2O + H^+	99.06871 + 18.01057 + 1.00783 = 118.08681	-4.23
173.09197	A_2pm (mesoDap) + H^+	172.08479 + 1.00783 = 173.09262	-3.76
204.08655	GlcNAc + H^+	203.07937 + 1.00783 = 204.08720	-3.18
290.17065	VA_2pm + H_2O + H^+	117.07898 + 172.08479 + 1.00783 = 290.17160	-3.27

(GMTripeptide + V) deacetylated

$[M+2H]^{2+}$ = 464.7214; Neutral mass = 927.4284; RT= 13.35 min; error ppm = -0.22

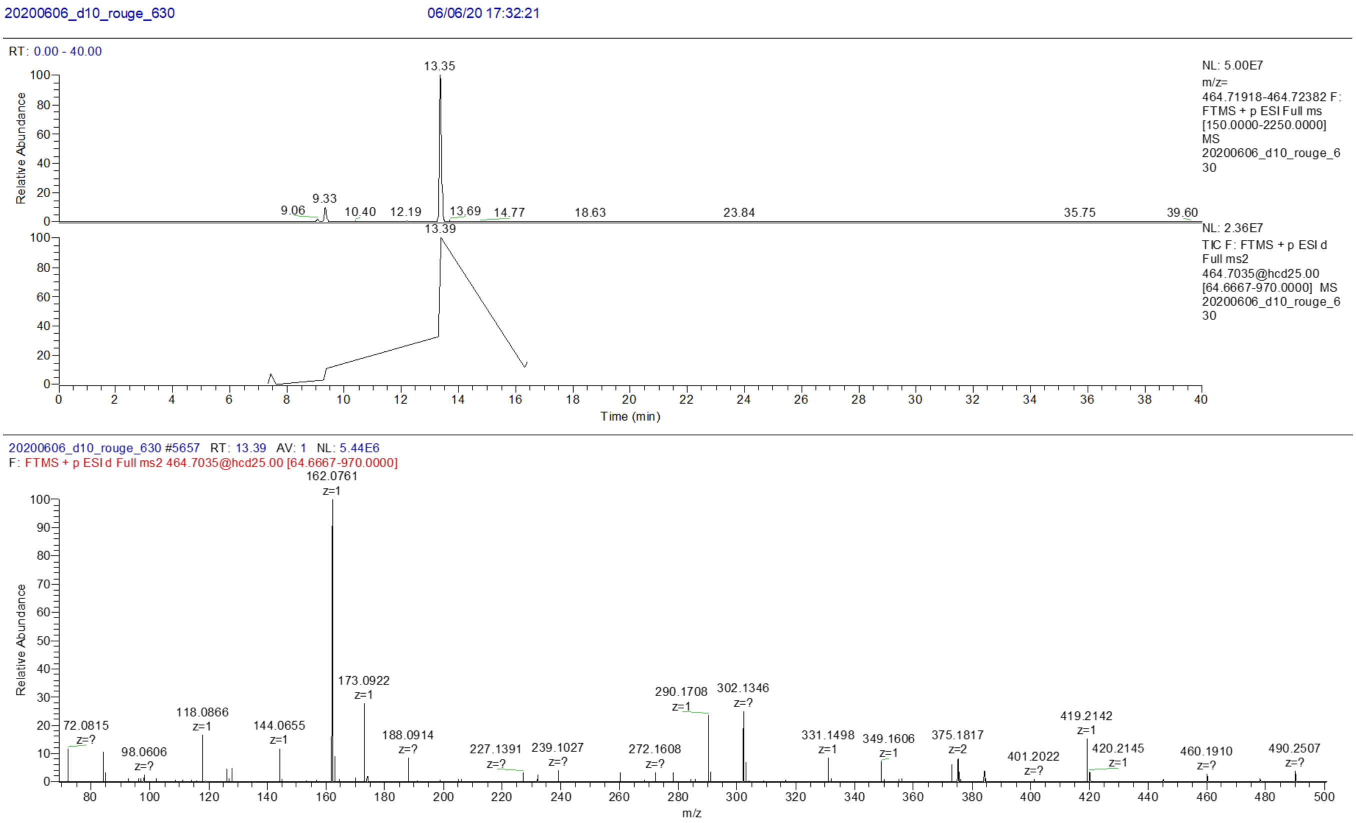

Figure 15: fragmentation MS/MS of m/z 464.7035 ion. New analysis 2020_06

GMTripeptide + V

$[M+2H]^{2+}$ = 485.7268; Neutral mass = 969.4390; RT= 14.76 min; error ppm = 0

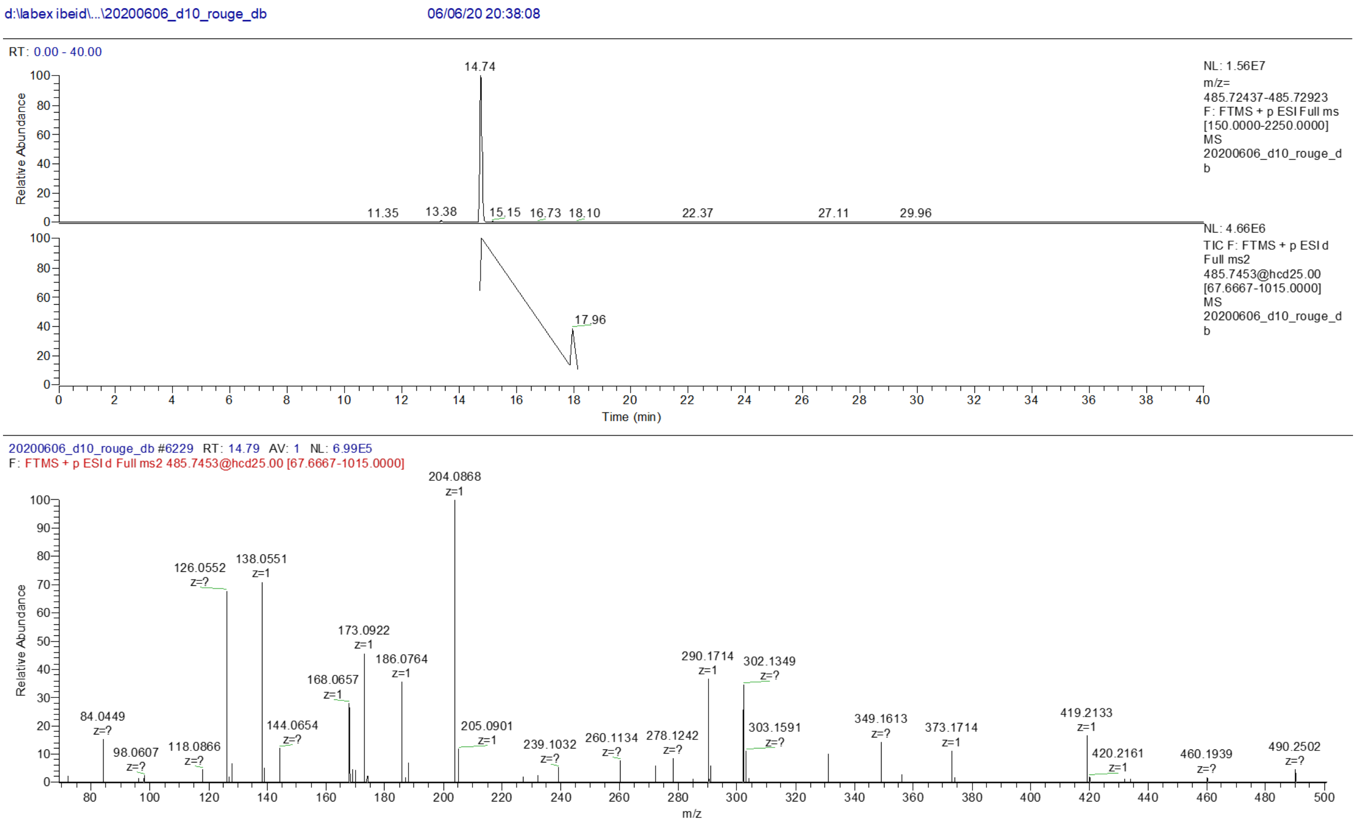

Figure 16: MS/MS fragmentation of m/z 485.7453. New analysis 2020_06

1.1.4 Modified alanine

GMTetrapeptide with modified alanine in 4th position on stem peptide; deacetylated

$[M+2H]^{2+}$ = 443.7162; Neutral mass = 885.4188; RT= 9.80 min; error ppm = 1.35

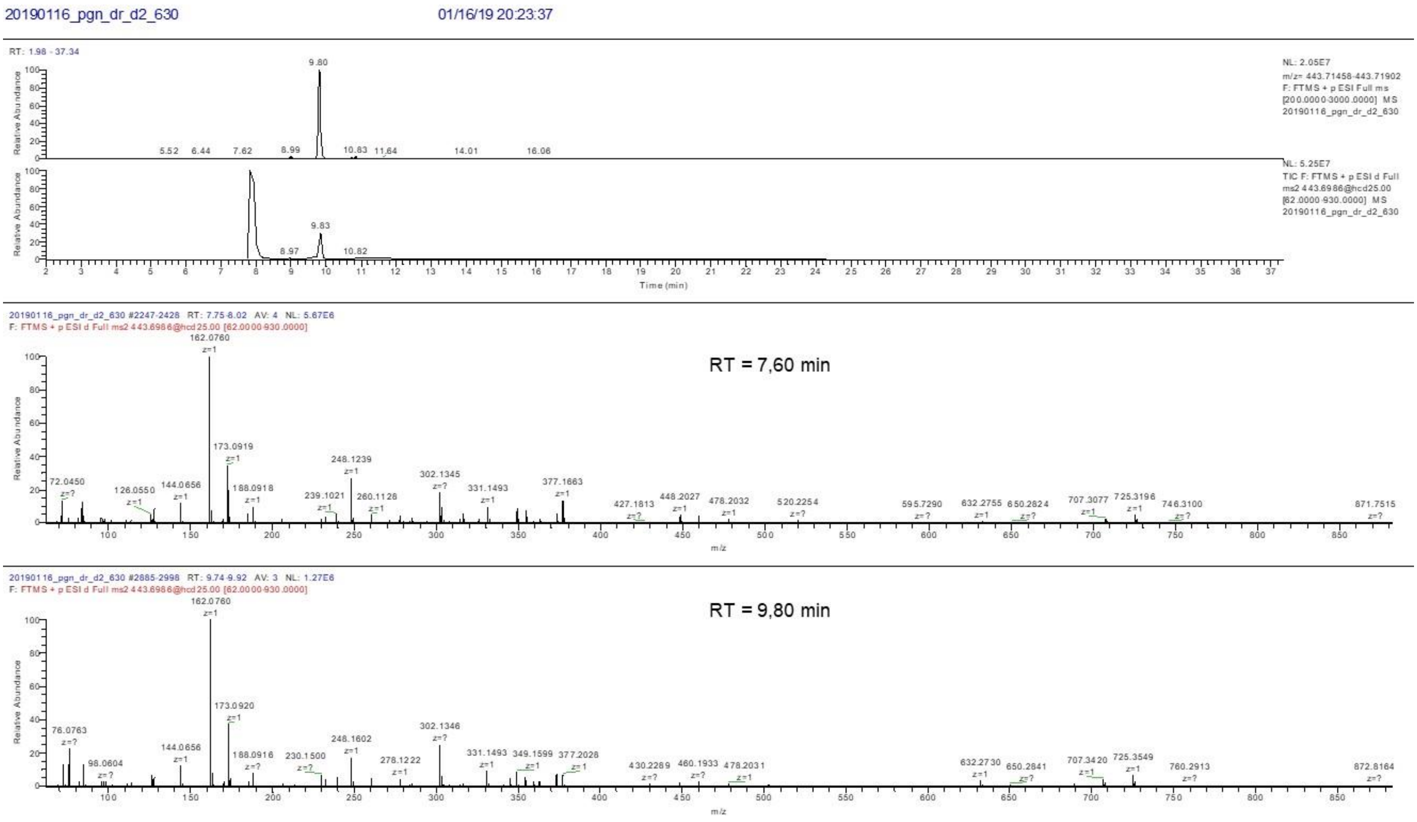

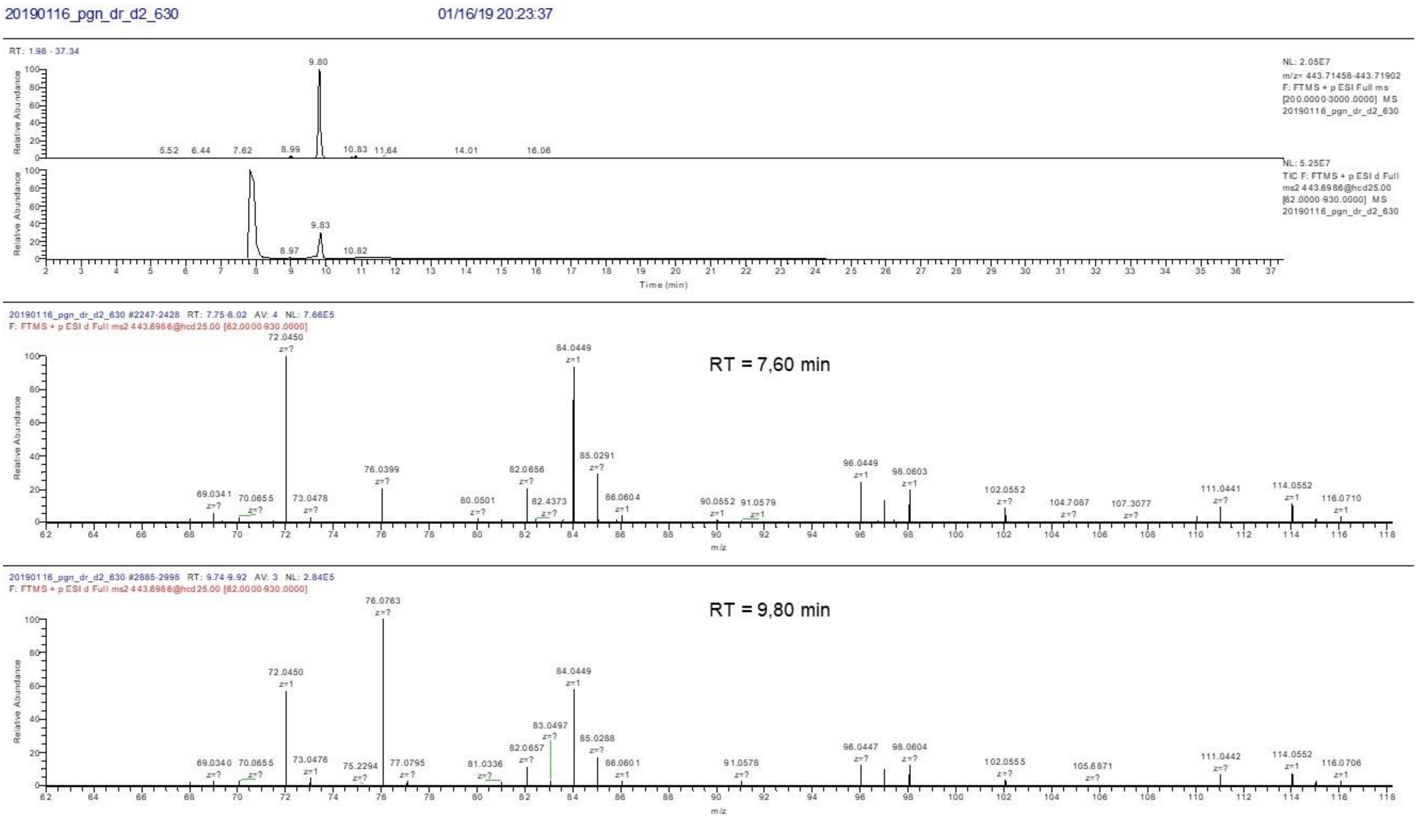

RT min	m/z obs. in MS/MS spectrum	residue	m/z th.	Error ppm
7.80	76.0399	Glycine + H_2O + H^+	57.02147 + 18.01057 + 1.00783 = 76.03987	0.39
	162.0760	Glucosamine + H^+	161.06881 + 1.00783 = 162.07664	-3.95
	248.1239	A_2pm (mesoDap) + Gly+ H_2O + H^+	172.08479 + 76.03987 = 248.12466	-3.06

9.80	76.0763	A* + H_2O + H^+	75.06841 + 1.00783 = 76.07624	0.79
	248.1602	A_2pm (mesoDap) + A*+ H_2O + H^+	172.08479 + 76.07624 = 248.16103	-3.34

In a second analysis :

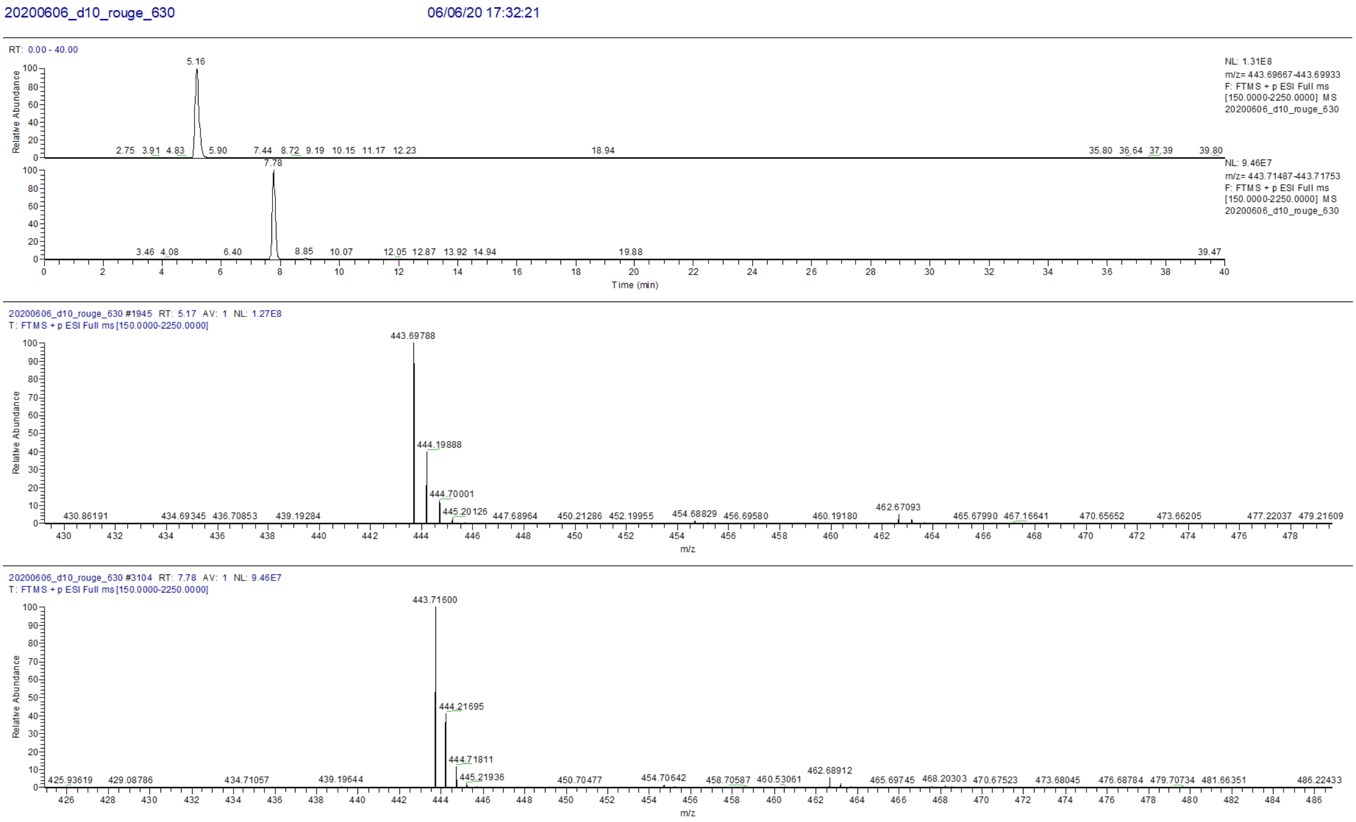

Figure 17: MS scan of m/z 443.6980 (GM3+Gly) and m/z 443.7162 (GM4 with Ala modified) in wt strain.

1.1.5 Gly and I/L

GMTripeptide (+Gly +I/L)

$[M+2H]^{2+}$ = 521.2457; Neutral mass =1040.4761; RT= 18.20 min; error ppm = 0.77

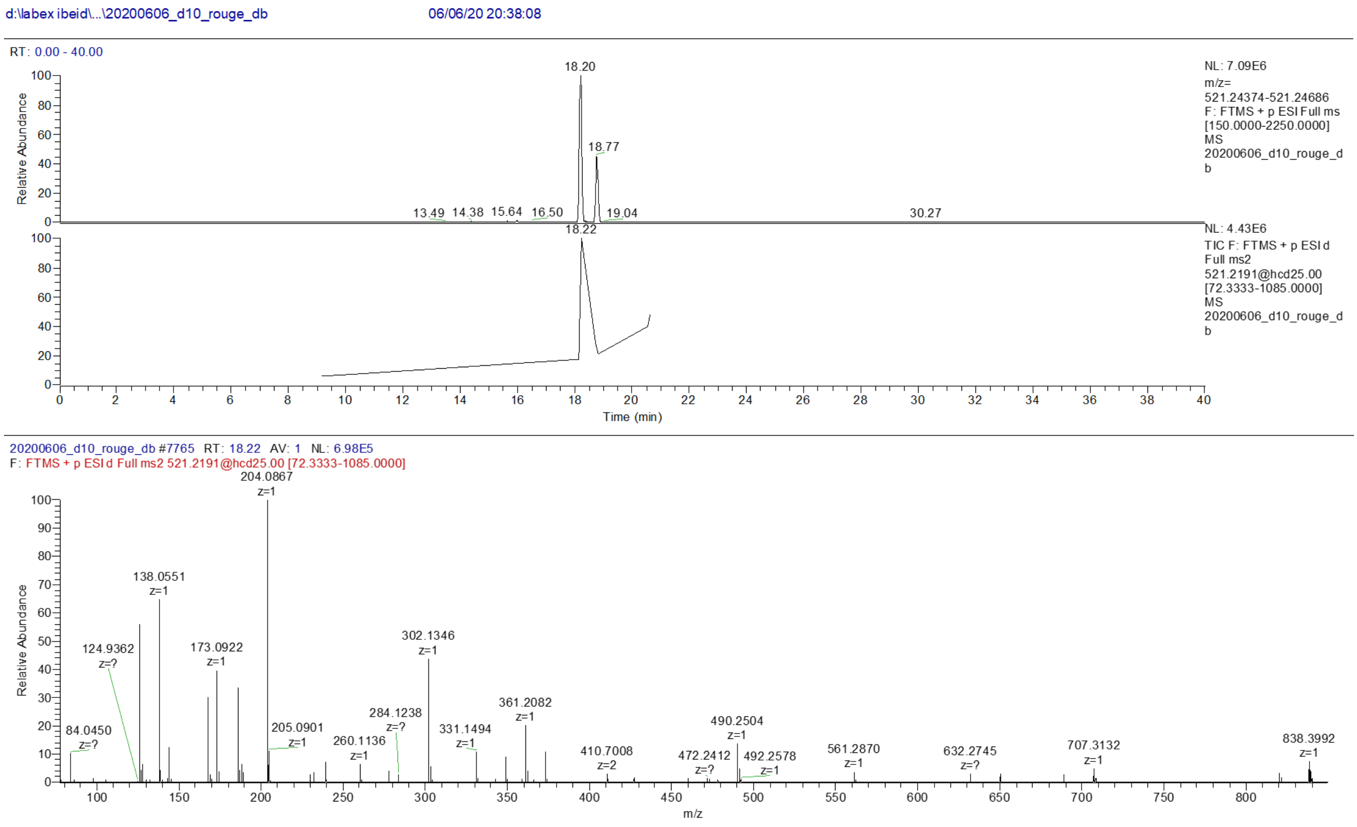

Figure 18: XIC and MS2 of Tri+Gly+I/L in double mutant. New Analysis 2020_06

m/z obs. in MS/MS spectrum	residue	m/z th.	Error ppm
86.09704	I/L immonium ion	86.09698	0.70
204.0867	GlcNAc + H^+	203.07937 + 1.00783 = 204.08720	-2.45
343.1975	A_2pm (mesoDap)+I/L+Gly+H^+	172.08479 +113.08406 + 57.02146+1.00783= 343.1981	-1.75
361.2082		343.19814+ 18.01057= 361.2087	-1.38
490.2504	EA_2pm (mesoDap)+I/L+Gly+H_2O+H^+	361.2087 + 129.04260 = 490.2513	-1.84

GMTripeptide (+Gly +I/L) deacetylated

$[M+2H]^{2+}$ = 500.2399; Neutral mass = 998.4656; RT= 16.55 min; error ppm = -0.40

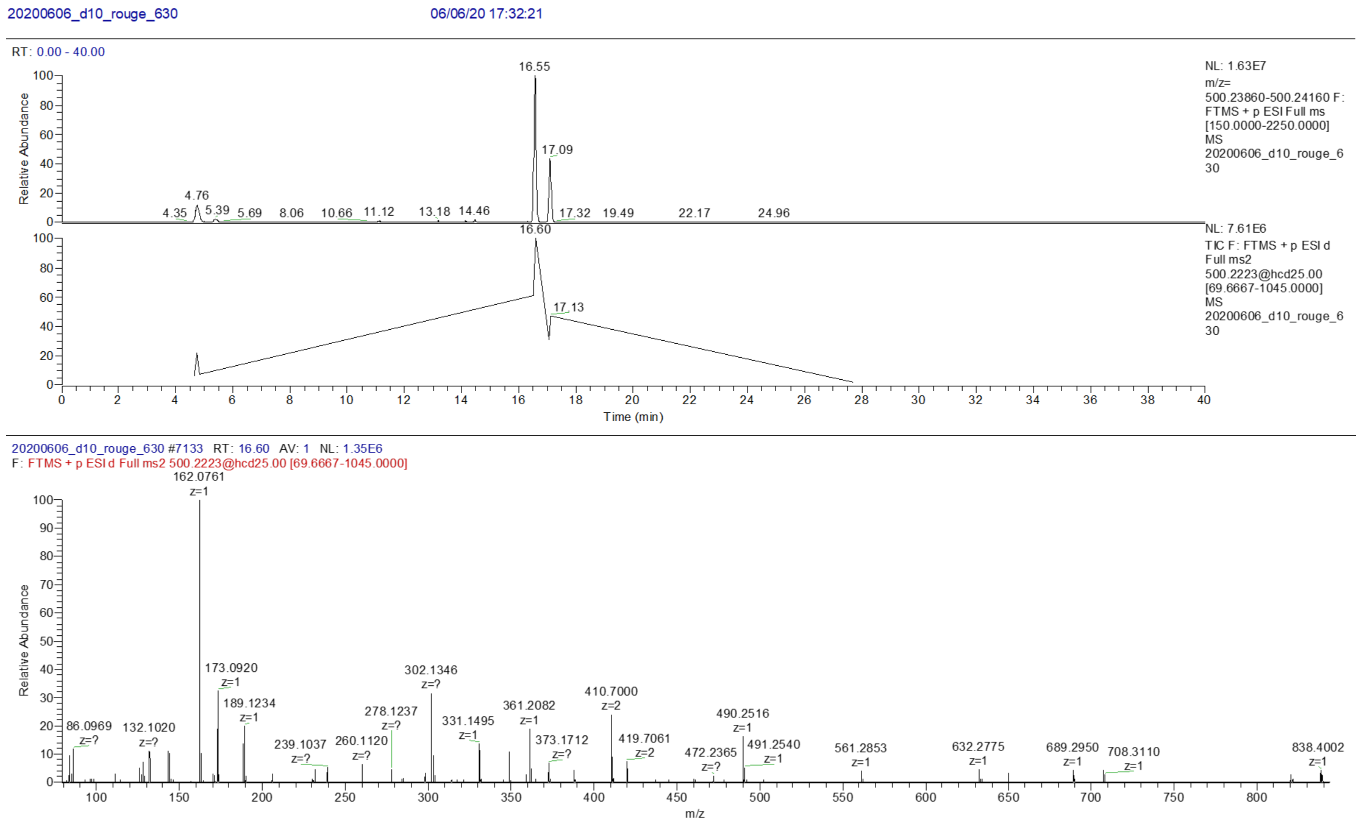

Figure 19 : XIC and MS2 of Tri+Gly+I/L deacetylated in wt strain. New Analysis 2020_06

m/z obs. in MS/MS spectrum	residue	m/z th.	Error ppm
86.0969	I/L immonium ion	86.09698	-0.93
162.0761	Glucosamine + H^+	161.06881 + 1.00783 = 162.07664	-3.33
230.1137	A_2pm (mesoDap)+Gly+H^+	172.08479 + 57.02146+1.00783= 230.1141	-1.74
361.2082		343.19814+ 18.01057= 361.2087	-1.38
490.2516	EA_2pm (mesoDap)+I/L+Gly+H_2O+H^+	361.2087 + 129.04260 = 490.2513	0.61

1.2 Regular Amino Acid

1.2.1 Lysine

Clarify ambiguities regarding GM3+K and GM3+K_02

$[M+2H]^{2+}$ = 500.2401; Neutral mass = 998.4656; RT= 4.70 and 5.35 min; error ppm = 0.60

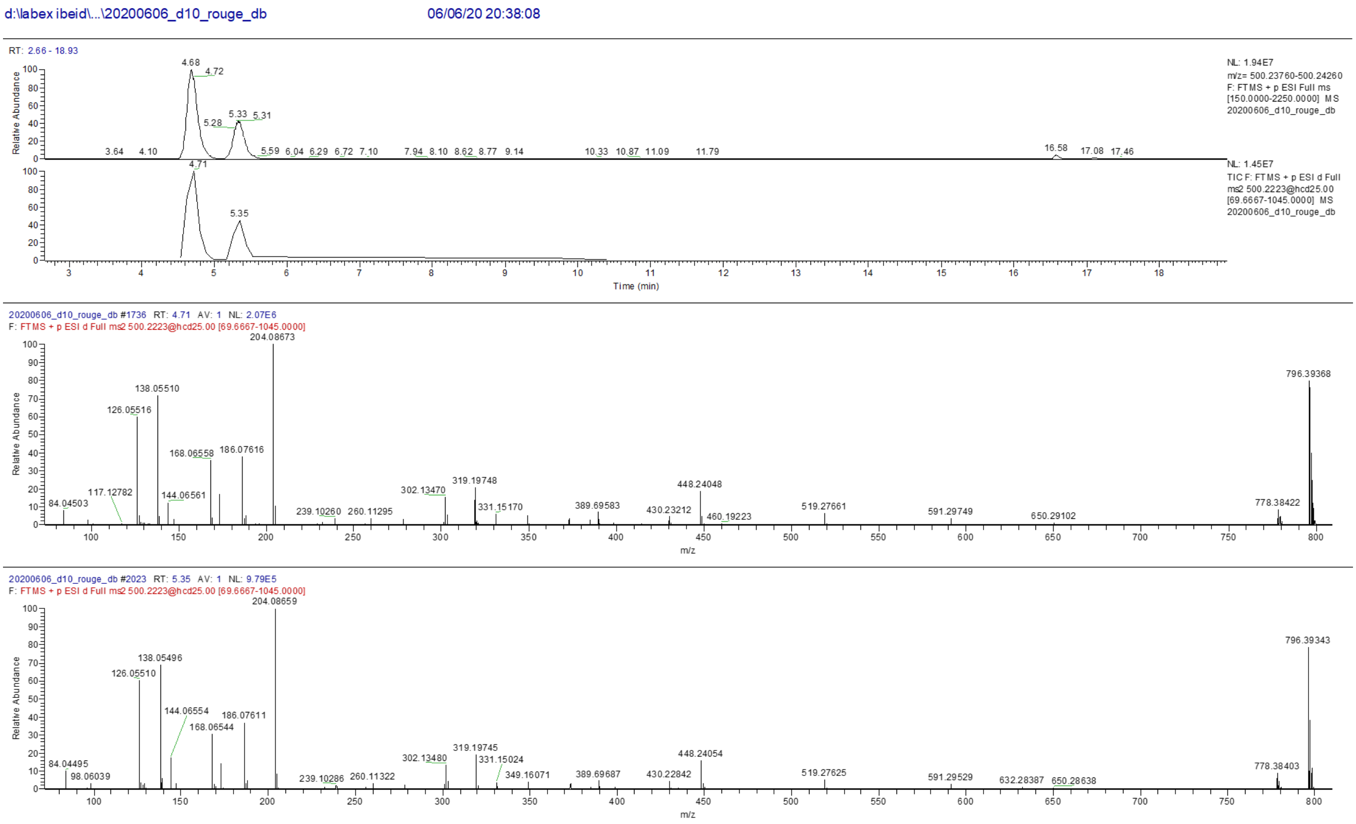

Figure 20: Double mutant sample. MS/MS fragmentation spectra of m/z 500.2401 at 4.71 and 5.35 min.

Unfortunately, no difference between the ion fragments was noticed in the double peak corresponding to *m/z 500.2401*. So, I consider that these peaks represent the same molecule GMTripeptide + K and I combined its areas.
