# Supplementary material for: Peptidoglycan analysis reveals that synergistic deacetylase activity in vegetative Clostridium difficile impacts the host response
Source: J Biol Chem. 2021 Jan 13;295(49):16785–96. doi: 10.1074/jbc.RA119.012442 (PMC7864072; doi:10.1074/jbc.RA119.012442)
Supplement: Supplementary file 1 [file mmc1.zip › 157925_2_supp_602953_qh86zk.pdf]

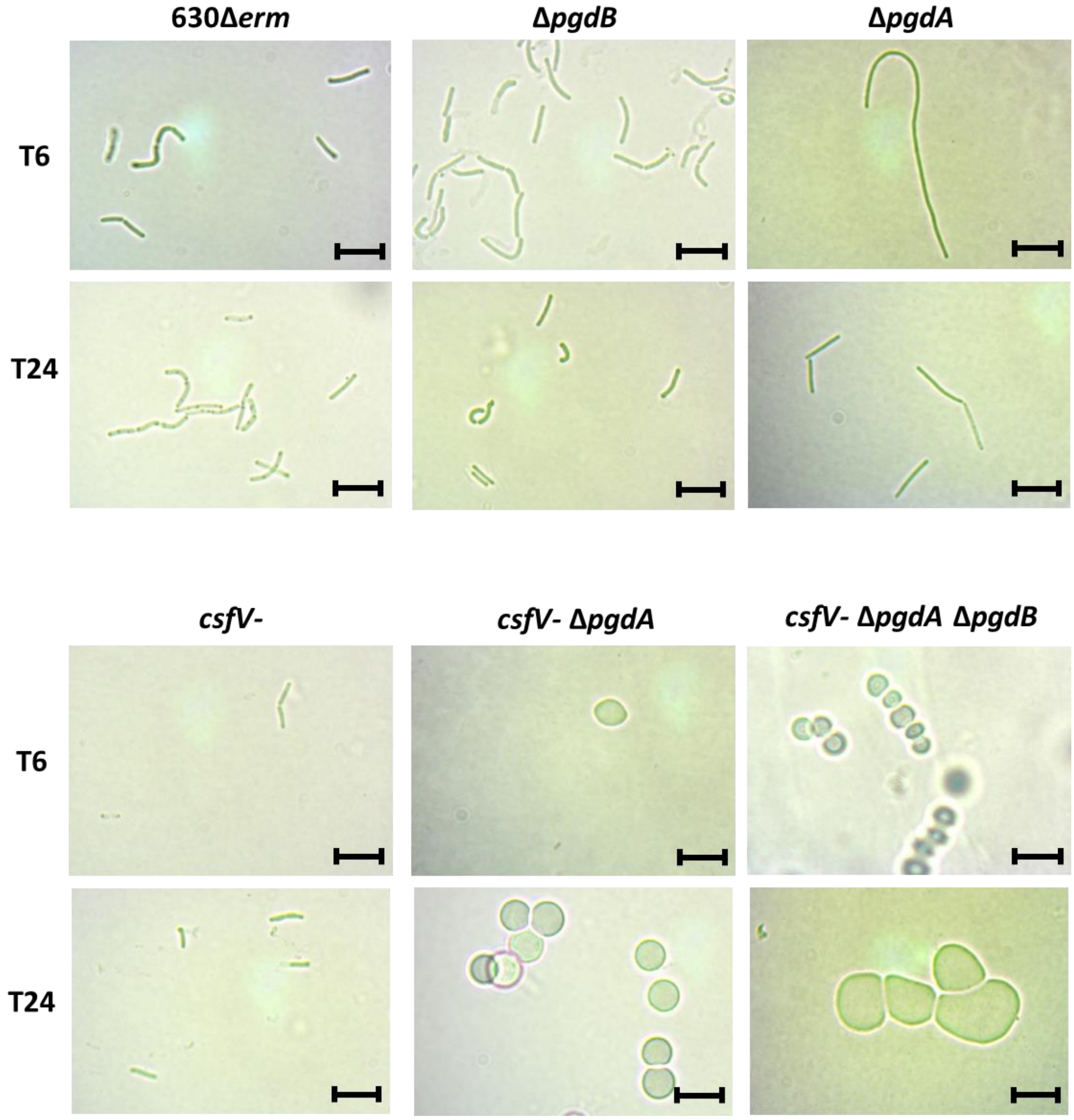

Figure S1. Lysozyme induces morphology defects in exponential and stationary phase.
Optical microscopy observations of fresh samples after 6 and 24 hours of growth in BHISG supplemented with 1 mg/mL lysozyme. Conventional optical microscopy observations were conducted using a Primo Star Zeiss microscope, with a mounted AxioCam ERc 5s camera. Cultures samples were spotted onto thin agar slices, and observed using oil immersion with the x100 objective. Scale: 10µm.

Figure S2

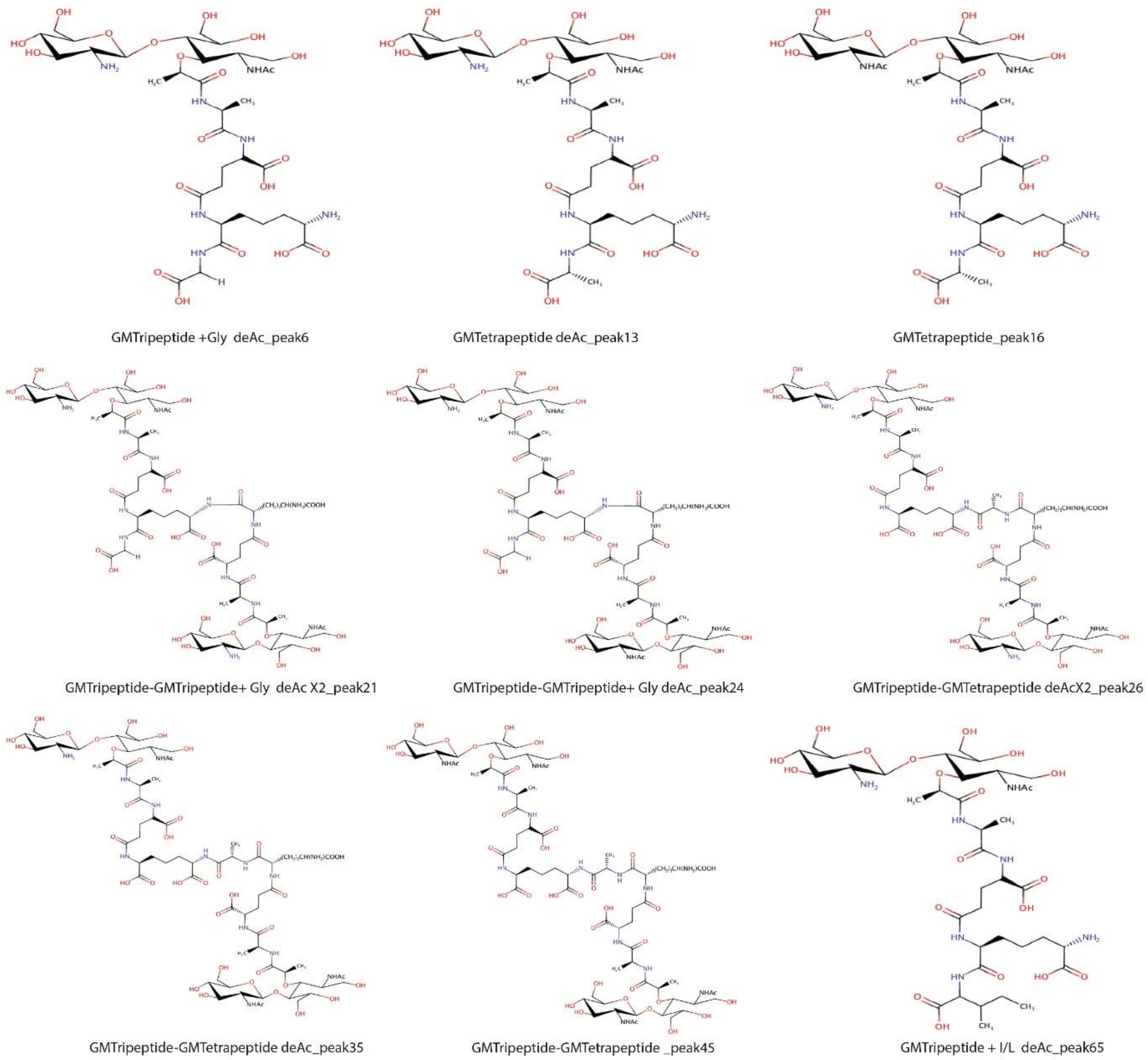

Figure S2. Structures of the main muropeptides identified in the peptidoglycan analysis
Structures of peaks 6,13,16,21,24,26,35,45 and 65 identified in Figure 3 and Table S2.

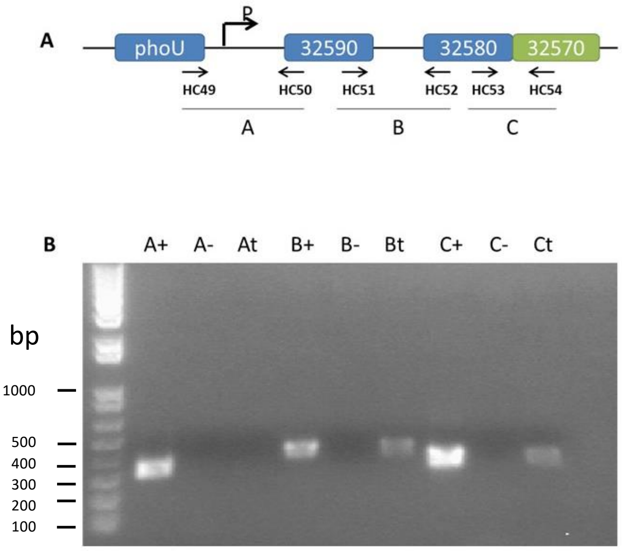

Figure S3. Identification of the *pgdB* promoter

(A) The genetic region of *pgdB* is represented with the primers designed to amplify the intergenic regions A, B and C. Each region was amplified using three samples: genomic DNA (+), RNA (-) and complementary DNA (t). (B) Agarose gel migration of the PCR, using 1kb Plus DNA ladder from Invitrogen. The base pairs are indicated (bp). Regions B and C show an amplification around 400 and 600bp respectively wen run with gDNA (lanes B+ and C+) and cDNA (lanes Bt and Ct), which suggests that both regions are transcribed in the bacteria. However, there is no amplification of intergenic region A when cDNA is used (lane At) which indicates that this region is not transcribed by the bacteria. We therefore used this region as the promoter of *pgdB*. Primers are described in the Table S3.

Protein sequence identity (%)							
	C. difficile **CD630_15220**	***E. faecalis*** **PgdA**	***C. difficile*** **CD630_32570**	***C. difficile*** **CD630_15560**	***B. anthracis*** **BA5436**	***B. anthracis*** **BA1977**	***B. anthracis*** **BA2944**
C. difficile **CD630_15220 (PgdA)**	/	33.80	25.91	24.90	25.23	27.85	26.78
E. faecalis **PgdA**	33.80	/	25.90	26.59	28.70	28.63	25.51
C. difficile **CD630_32570 (PgdB)**	25.91	25.90	/	43.82	30.96	36.68	32.57
C. difficile **CD630_15560 (PdaV)**	24.90	26.59	43.82	/	33.05	33.07	33.33
B. anthracis **BA5436**	25.23	28.70	30.96	33.05	/	38.49	37.45
B. anthracis **BA1977**	27.85	28.63	36.68	33.07	38.49	/	66.05
B. anthracis **BA2944**	26.78	25.51	32.57	33.33	37.45	66.05	/

Table S1. Percent Identity Matrix

Identity matrix of protein sequences of know peptidoglycan N-deacetylases from *Bacillus anthracis* [7], *Enterococcus faecalis* [6] and *Clostridium difficile*, built with Clustal Omega multiple sequences alignment [21].

Peak	Muropeptides	RT	Formula	m/z obs.	m/z th.	Percentage of all the muropeptide					
						630Δ*erm*	Δ*pgdB*	Δ*pgdA*	*csfV-*	*csfV::erm* Δ*pgdA*	*csfV::erm* Δ*pgdA* Δ*pgdB*
1	Disaccharide	4.1	C19H34N2O13	499.2061	499.2134	0.17	0	0	0	3.88	2.81
2	GMTripeptide + K deAc	4.85	C38H68N8O20	479.2350	479.2348	0.26	0	0.18	0	Traces	Traces
3	GMTripeptide + K deAc_02	5.7	C38H68N8O20	479.2350	479.2348	0.11	0.12	0.11	0.14	Traces	Traces
4	GMTripeptide deAc	6.4	C32H56N6O19	415.1871	415.1864	1.59	0	0	0	0	0
5	GMTripeptide + K	6.8	C40H70N8O21	500.2404	500.2401	Traces	0	0	0	0.51	0.41
6	GMTripeptide +Gly deAc	7.8	C34H59N7O20	443.698	443.6980	5.90	5.67	5.31	5.72	0.29	0.21
7	GMTripeptide + K_02	7.8	C40H70N8O21	500.2404	500.2401	0	0	0	0	0.24	0.20
8	GMTripeptide	8.1	C34H58N6O20	436.1927	436.1926	0	0	0.43	0	1.17	1.33
9	Tetrasaccharide deAc	9	C36H62N4O24	468.1964	468.1950	0.07	0	0	0	0.08	0.06
10	GMTripeptide +Gly	9.05	C36H61N7O21	464.7035	464.7033	0	0	1.07	0	3.90	2.42
11	GMDipeptide deAc	9.7	C25H44N4O16	657.2825	657.2811	1.50	1.54	1.80	1.71	0	0
12	GMTetrapeptide A* (C-ter) deAc	9.8	C35H63N7O19	443.7161	443.7162	1.10	1.11	0.80	1.06	0.01	0.04
13	GMTetrapeptide deAc	10.1	C35H61N7O20	450.7057	450.7059	25.80	25.90	17.53	26.82	0.81	1.02
14	GMTetrapeptide A* (C-ter)	10.7	C37H65N7O20	464.7212	464.7215	0.03	0.04	0.37	0.04	0.53	0.79
15	GMDipeptide	10.8	C27H46N4O17	699.293	699.2931	0	0.07	0.74	0	2.64	2.32
16	GMTetrapeptide	11.05	C37H63N7O21	471.7111	471.7102	0.35	0.54	5.46	0.72	13.31	13.23
17	Tetrasaccharide-Tetra deAcX2	11.6	C52H89N9O31	446.1973	446.1961	0.11	0.24	0.07	0.36	0	0
18	Tetrasaccharide-AE deAcX2	12	C42H72N6O27	547.231	547.2296	0.20	0.20	0	0.25	0	0
19	Tetrasaccharide-Tetra deAc	12.3	C54H91N9O32	689.7972	689.7958	0.05	0.08	0.34	0.10	0	0
20	GMTetrapeptide-GMTetrapeptide A* deAc	12.55	C72H124N14O39	905.4153	905.4148	0.06	0.03	0	0	0	0
21	GMTripeptide-GMTripeptide+ Gly deAc X2	13	C66H113N13O38	848.8729	848.8728	2.32	2.53	1.53	2.57	0	0
22	GMTripeptide-GMTripeptide deAcX2	13.3	C64H110N12O37	547.244	547.2431	1.01	1.12	0.72	1.12	0	0
23	GMTetrapeptide-GMTripeptide + G deAcX2	13.6	C69H118N14O39	589.9301	884.3913	0.34	0.36	0.21	0.34	0	0
24	GMTripeptide-GMTripeptide+ Gly deAc	13.7	C68H115N13O39	869.8782	869.8780	0	0.40	3.08	0.55	0.45	0.47
25	GMTripeptide-GMTetrapeptide A* (C-ter) deAcX2	14	C67H117N13O37	848.8906	848.8910	0.18	0.17	0.09	0.16	0	0
26	GMTripeptide-GMTetrapeptide deAcX2	14.2	C67H115N13O38	570.923	570.9228	29.80	31.62	14.95	28.37	0.08	Traces
27	GMTripeptide-GMTripeptide + Gly	14.3	C70H117N13O40	890.8831	890.8833	0	0	0.73	0	8.26	5.86
28	GMTetrapeptide-GMTripeptide + G deAc	14.4	C71H120N14O40	905.3969	905.3966	0	0	0	0	0.11	0.08
29	Tetrasaccharide-Tetra+GMTripeptide deAcX3_01	14.4	C84H143N15O49	537.4868	537.4863	0.08	0.16	0.04	0.26	0	0
30	GMTripeptide-GMTripeptide	14.5	C68H114N12O39	862.3725	862.3726	0	0	0.18	0	1.79	2.29
31	GMTripeptide-GMTripeptide A* (C-ter) deAcX2_02	14.5	C67H117N13O37	848.8906	848.8910	0.15	0.14	0.07	0.14	0	0

32	GMTetrapeptide-GMTetrapeptide A* deAcX2	14.6	C70H122N14O38	884.4089	884.4095	Traces	0	0	0	0	0
33	GMTripeptide-GMTetrapeptide A* (C-ter) deAc	14.7	C69H119N13O38	869.8959	869.8962	0.06	0.09	0.44	0.09	Traces	Traces
34	GMTetrapeptide-GMTetrapeptide deAcX2	14.95	C70H120N14O39	594.6016	594.6018	9.93	10.25	2.87	7.34	0	0
35	GMTripeptide-GMTetrapeptide deAc	15	C69H117N13O39	876.8855	876.8867	1.85	2.31	12.68	2.21	1.25	1.99
36	GMTetrapeptide-GMTripeptide + G	15	C73H122N14O41	926.402	926.4019	0	0	0.19	0	2.46	1.67
37	GMTripeptide-GMTripeptide-GMTripeptide + G deAcX3	15	C98H167N19O56	627.5273	836.3674	0.31	0.49	0.19	0.53	0	0
38	GMTripeptide + V deAc	15.2	C37H65N7O20	464.7214	464.7215	0.47	0.29	0.38	0.31	0.02	0.03
39	GMTripeptide-GMTetrapeptide A* (C-ter) deAc_02	15.25	C69H119N13O38	869.8959	869.8962	0.05	0.06	0.33	0	Traces	Traces
40	GMTetrapeptide-GMTetrapeptide A* deAcX2_02	15.25	C70H122N14O38	884.4089	884.4095	Traces	0	0	0	0	0
41	Tetrasaccharide-Tetra+GMTripeptide deAcX2_01	15.25	C86H145N15O50	730.3171	730.3161	0.03	0.06	0.20	0.10	0	0
42	GMTripeptide-GMTetrapeptide A* (C-ter)	15.3	C71H121N13O39	890.9014	890.9015	0	0	0.17	0	1.13	1.75
43	GMTripeptide-GMTetrapeptide-GMTripeptide + G deAcX3	15.45	C101H172N20O57	860.0484	860.0465	0.10	0.16	0.07	0.17	0	0
44	GMTetrapeptide-GMTetrapeptide deAcX2_02	15.5	C70H120N14O39	594.6016	594.6018	0	0	1.36	3.44	0	0
45	GMTripeptide-GMTetrapeptide	15.7	C71H119N13O40	897.8908	897.8911	0	0	3.50	0	25.88	25.93
46	GMTetrapeptide-GMTetrapeptide deAc	15.75	C72H122N14O40	912.4045	912.4059	0	0	0	0	0.45	0
47	GMTripeptide-GMTetrapeptide A* (C-ter) _02	15.87	C71H121N13O39	890.9014	890.9015	0	0	0.14	0	1.15	1.56
48	GMTetrapeptide-GMTetrapeptide A*	16.02	C74H126N14O40	926.4201	926.4201	0	0	0.09	0	0	0.58
49	GMTripeptide-GMTripeptide-GMTetrapeptide deAcX3	16.15	C99H169N19O56	841.0404	841.0393	1.42	1.59	0.48	1.68	0	0
50	GMTripeptide + V	16.25	C39H67N7O21	485.7268	485.7268	0	0	0.09	0	0.43	0.57
51	GMTetrapeptide-GMTetrapeptide	16.4	C74H124N14O41	933.4097	933.4097	0	0	1.74	0	8.81	8.10
52	GMTripeptide-GMTetrapeptide-GMTetrapeptide deAcX3	16.55	C102H174N20O57	864.7197	864.7183	0.87	0.83	0.23	0.83	0	0
53	GMTetrapeptide-GMTetrapeptide A*_02	16.64	C74H126N14O40	926.4201	926.4201	0	0	0.08	0	0	0.55
54	GMTripeptide-GMTripeptide-GMTripeptide + G	16.75	C104H173N19O59	878.3784	878.3780	0	0	0	0	0.53	0.47
55	GMTripeptide-GMTripeptide-GMTetrapeptide deAcX2	16.85	C101H171N19O57	855.0426	855.0428	0.17	0.35	1.35	0.51	0	0
56	GMTetrapeptide-GMTetrapeptide-GMTetrapeptide deAcX3	17.05	C105H179N21O58	888.3992	888.3974	0.07	0.07	0	0.07	0	0
57	GMTripeptide-GMTetrapeptide-GMTripeptide + G	17.3	C107H178N20O60	902.0576	902.0570	0	0	0	0	0.31	Traces
58	GMTripeptide-GMTetrapeptide-GMTetrapeptide deAcX2	17.31	C104H176N20O58	878.7214	878.7207	0.18	0.32	1.03	0.29	0	0
59	GMTetrapeptide-GMTetrapeptide-GMTetrapeptide deAcX3_02	17.4	C105H179N21O58	888.3988	888.3974	0.05	0.05	0	0.04	0	0
60	GMTetrapeptide-GMTetrapeptide-GMTetrapeptide deAcX2	17.9	C107H181N21O59	902.4008	902.4009	0	0	0.13	0	0	0
61	GMTripeptide-GMTripeptide-GMTetrapeptide	18	C105H175N19O59	883.0499	883.0499	0	0	0	0	2.00	2.40
62	GMTripeptide-GMTetrapeptide-GMTetrapeptide	18.5	C108H180N20O60	906.7294	906.7289	0	0	0	0	1.71	1.77
63	GMTripeptide+I/L+Gly deAc	18.61	C40H70N8O21	500.2404	500.2401	0.34	0.18	0.24	0.20	0	0

64	GMTetrapeptide-GMTetrapeptide-GMTetrapeptide	18.94	C111H185N21O61	930.4081	930.4079	0	0	0.04	0	0	0
65	GMTripeptide + I/L deAc	19.05	C38H67N7O20	471.7292	471.7293	3.34	2.37	3.14	2.44	0.21	0.39
66	GMTripeptide +I/L+Gly deAc_02	19.22	C40H70N8O21	500.2404	500.2401	0.23	0.11	0.16	0.12	0	0
67	GMTripeptide+I/L+Gly	19.77	C42H72N8O22	521.2457	521.2453	0	0	0.06	0	0.16	0.12
68	GMTripeptide + I/L	20.3	C40H69N7O21	492.7348	492.7346	0.05	0.04	0.70	0.06	2.45	3.03
69	GMTripeptide+I/L+Gly	20.4	C42H72N8O22	521.2457	521.2453	0	0	0.04	0	0.09	0.07
70	GMTripeptide + F deAc	20.75	C41H65N7O20	488.7218	488.7215	4.02	3.44	4.40	3.80	0.26	0.42
71	GMTripeptide-GMTripeptide+ I/L deAcX2	21.2	C70H121N13O38	584.939	584.9385	2.25	1.77	1.40	1.90	Traces	Traces
72	GMTetrapeptide-GMTripeptide+ I/L deAcX2	21.9	C73H126N14O39	912.4243	912.4226	0.10	0.07	0.04	0.06	0	0
73	GMTripeptide + F	22.1	C43H67N7O21	509.7275	509.7268	0.06	0.06	0.92	0.09	2.87	3.53
74	GMTripeptide-GMTripeptide + I/L deAc	22.25	C72H123N13O39	598.9424	598.9420	0.03	0.03	0.29	0.05	0.04	Traces
75	GMTripeptide-GMTripeptide + F deAcX2	22.3	C73H119N13O38	596.267	596.2666	2.73	2.87	2.26	3.13	0	0
76	GMTetrapeptide-GMTripeptide + F deAcX2	22.8	C76H124N14O39	929.4167	929.4148	0.11	0.09	0.07	0.10	0	0
77	GMTripeptide-GMTripeptide+ I/L	23	C74H125N13O40	918.9151	918.9146	0	0	0.26	0	3.33	4.24
78	GMTetrapeptide-GMTripeptide+ I/L deAc	23	C75H128N14O40	933.4302	933.4279	0	0	0.22	0	0	0
79	GMTripeptide-GMTripeptide+ F deAc	23.45	C75H121N13O39	914.9022	914.9015	0	0	1.33	0	0.20	0.24
80	GMTetrapeptide-GMTripeptide+ I/L	23.7	C77H130N14O41	954.4345	954.4332	0	0	0.10	0	1.03	1.17
81	GMTetrapeptide-GMTripeptide + F deAc	24	C78H126N14O40	950.4227	950.4201	0	0	0.31	0	0	0
82	GMTripeptide-GMTripeptide + F	24.25	C77H123N13O40	935.9077	935.9068	0	0	0.36	0	3.86	4.55
83	GMTetrapeptide-GMTripeptide + F	24.8	C80H128N14O41	971.4248	971.4254	0	0	0.12	0	1.29	1.33

Table S2. Muropeptides detected in the peptidoglycan analysis of the 630Δ*erm* parental strain and the mutants
RT, retention time; ND, not detected in the profile; GM, Glc*N*Ac-*N*-acetylmuramitol (reduced Glc*N*Ac-Mur*N*Ac); Tri, disaccharide-tripeptide; Tetra, disaccharide-tetrapeptide; deAc, *N*-deacetylation of the glucosamine; deAcX2, number of *N*-deacetylated glucosamine; K, Lysine; Gly, Glycine; A*, modified Alanine; V, Valine; I/L, Isoleucine or Leucine; F, Phenylalanine (see Supplementary data_Structural identification for more details). Tri and tetra contain E-mDAP. Muropeptides marked with '_02' refer to the second peak identified for a single muropeptide. The presence of a double peak for a single muropeptide may be due to the crosslink arrangement inside the dimers. The exact mass experimentally observed (m/z obs.) versus the theoretical exact mass (m/z th.) are provided. Percentage of each peak was calculated using the extracted ion current chromatogram, and defined as the ratio of the peak area over the sum of areas of all the peaks identified in the table times 100. Results show the analysis of a single biological replicate representative of three independent biological replicates. The full raw data of every replicate has been deposited on MassIVE, available under https://massive.ucsd.edu/ProteoSAFe/private-dataset.jsp?task=b10c92db58274bb9abe84a8a170477e9 (doi :10.25345/C51N1R).

**Name**	**Characteristics**	**Reference**
**Bacterial strains**		
Escherichia coli		
TG1	*E. coli* k12 (F', *tra* D36, *lacIq*, Δ*lacZ*, MIS, *pro* A+B+/SupE, Δ(*hdsM-mcrB*))	Laboratory
HB101pRK24	*E. coli* (pRK24) (F - Δ(*gpt-proA*) 62 Leu B6 *gln* V44 *ara*-14 *gal*K2 *lac*Y1 Δ(*mcrC-mrr*) *rps* L20 (*srt*^{r}) *xyl*-5 *mlt*-1 *rec* A13, pRK24)	Laboratory
Clostridium difficile		
630	Reference strain, obtained from a pseudomembrane clinical isolate	[31]
630Δ*erm*	Erythromycin sensitive 630 derived strain	[30]
Δ*pgdA*	ΔCD630_15220 derivative of 630Δ*erm* strain	This work
Δ*pgdB*	ΔCD630_32570 derivative of 630Δ*erm* strain	This work
csfv-	*csfV* ClosTron insertion derivative of 630Δ*erm* strain	[24]
csfV::erm Δ*pgdA*	ΔCD630_15220 derivative of the 630Δ*erm csfV::erm* strain (double mutant)	This work
csfV::erm Δ*pgdA* Δ*pgdB*	ΔCD630_32570 derivative of the 630Δ*erm csfV::erm* Δ*pgdA* (triple mutant)	This work
630Δ*erm*(pCH31)	630Δ*erm* carrying the plasmid for P*pgdB-gusA* expression	This work
630Δ*erm*(pCH32)	630Δ*erm* carrying the plasmid for P*pgdA-gusA* expression	This work
630Δ*erm*(pCH56)	630Δ*erm* carrying the plasmid for P*pdaV-gusA* expression	This work
csfv-(pCH31)	*csfv*- carrying the plasmid for P*pgdB-gusA* expression	This work
csfv-(pCH32)	*csfv*- carrying the plasmid for P*pgdA-gusA* expression	This work
csfv-(pCH56)	*csfv*- carrying the plasmid for P*pdaV-gusA* expression	This work
Δ*pgdA*(pCH31)	Δ*pgdA* carrying the plasmid for P*pgdB-gusA* expression	This work
Δ*pgdA*(pCH32)	Δ*pgdA* carrying the plasmid for P*pgdA-gusA* expression	This work
Δ*pgdA*(pCH56)	Δ*pgdA* carrying the plasmid for P*pdaV-gusA* expression	This work
Δ*pgdA*(pRPF185)	Δ*pgdA* strain carrying the empty plasmid pRPF185 for P_{tet} expression	This work
Δ*pgdA*(pCH70)	Δ*pgdA* strain carrying the P_{tet} plasmid for PgdB expression also named P_{TET}-*pgdB*	This work
Δ*pgdA*(pCH71)	Δ*pgdA* strain carrying the P_{tet} plasmid for PgdA expression also named P_{TET}-*pgdA*	This work
csfV::erm Δ*pgdA*(RPF185)	*csfV::erm* Δ*pgdA* strain carrying the empty plasmid pRPF185 for P_{tet} expression	This work
csfV::erm Δ*pgdA*(pCH71)	*csfV::erm* Δ*pgdA* strain carrying the P_{tet} plasmid for PgdA expression	This work
**Plasmids and vectors**		
pBLUNT	Linear cloning vector, blunt extremities, lacAα-ccdB, , kanamycin resistance	Invitrogen
pMTL-SC7315	Circular cloning vector, 6000 nucleotides, *catP, codA,*	[40]
pMTL-84151	Circular cloning vector, 6300 nucleotides, *catP*, α*LacZ*.	[41]
pRPF185	Circular cloning vector, 9000 nucleotides, *catP*, P_{tet}, *gusA*	[36]
pCH11	pMTLSC7315 derived plasmid for *pgdB* deletion	This work
pCH28	pMTLSC7315 derived plasmid for *pgdA* deletion	This work
pCH31	pMTL84151 derived plasmid for P_{pgdB} β-glucuronidase assay	This work
pCH32	pMTL84151 derived plasmid for P_{pgdA} β-glucuronidase assay	This work
pCH56	pMTL84151 derived plasmid for P_{csfV} β-glucuronidase assay	This work
pCH70	pRPF185 derived plasmid for P_{tet} inducible *pgdB* complementation	This work
pCH71	pRPF185 derived CPEC assembly for P_{tet} inducible *pgdA* complementation	This work

Table S3. Strains and plasmids used in the study.

Name	5' Sequence	3' Sequence	Use
**General use**			
M13F	/	tgtaaaacgacggccagt	Cloning screening
M13R	/	caggaaacagctatgacc	Cloning screening
TC157	/	gcagccagaagccatcgattacaaacgttg	pMTLSC7315 screening
VP52	/	GTTGACATTATATCATTGATAGAGTTATTTG	pRPF185 screening
tral for	/	CGTGATTGCCAAGCACGTCCCCATG	pRPF185 screening
**β-glucuronidase assay plasmids**			
HC87	ccatatgaccatgattacgaattcgagctc	GGTTGAGATAGGATAAAAAACTGC	CD630_32570 β-glucuronidase assay
HC88	tataaggtgctttttaggaggtggaact	ATGTTACGTCCTGTAGAAACCCCAACCCG	
HC89	GCAGTTTTTTATCCTATCTCAACC	gagctcgaattcgtaatcatggtcatatgg	
HC90	CGGGTTGGGGTTTCTACAGGACGTAACAT	agttccacctcctaaaaagcaccttata	
HC91	ccatatgaccatgattacgaattcgagctc	GTATTTTTTCAATAAAGCCCCCTCC	CD630_15220 β-glucuronidase assay
HC92	cgacaaaagggggaatgctgtaatt	ATGTTACGTCCTGTAGAAACCCCAACCCG	
HC93	GGAGGGGGCTTTATTGAAAAAATAC	gagctcgaattcgtaatcatggtcatatgg	
HC94	CGGGTTGGGGTTTCTACAGGACGTAACAT	aattacagcattcccccttttgtcg	
TC214	GAAAAACCGCAGCAGGGAGGCAAACAATGA	ggatcctctagagtcgacgtcacgcgtcc	β-glucuronidase assay
TC217	ggacgcgtgacgtcgactctagaggatcc	TCATTGTTTGCCTCCCTGCTGCGGTTTTTC	
HC231	ccatatgaccatgattacgaattcgagctc	GAGACTGTCTCAAATAGAGATAAGTC	*csfV* β-glucuronidase assay
HC232	/	ATGTTACGTCCTGTAGAAACCCC	
HC233	GGGGTTTCTACAGGACGTAACAT	cgttcgacctccaatattactatatttttac	
84151A	/	gagctcgaattcgtaatcatggtcatatgg	
**Mutant construction**			
HC5	GGATCC	gatgaagtcaaaagaccaataatatctg	CD630_32570 (*pgdB*) deletion DNA amplification
HC6	cttgccagaaatttttaggtgttatattttc	cccccatatctcccttttataaaagcataag	
HC7	cttatgcttttataaaagggagatatggggg	gaaaatataacacctaaaaatttctggcaag	
HC8	CTCGAG	ctatttcctctgtatatgtgaccttttttc	
HC51	/	aaaagccctgtattcaactatcatc	CD630_32570 (*pgdB*) deletion Allelic exchange screening
HC66	/	cgtgttcttcatgttcaacttctgcatctg	
HC67	/	attaattttatattatccttacagtatacc	
HC68	/	gagtagaatctttaggagaaaaagaagaag	
HC9	GGATCC	ctgcagttatgattgcaacaaatatcaag	CD630_15220 (*pgdA*) deletion DNA amplification
HC10	gagtataaaatgtacattcaataaacttgc	cagcattcccccttttgtcgaattgtatc	
HC11	gatacaattcgacaaaagggggaatgctg	gcaagtttattgaatgtacattttatactc	
HC12	CTCGAG	gaaagagttaagggaaaagcttattaaatc	
HC72	/	cataatacataatttatttgattaattagg	CD630_15220 (*pgdA*) deletion Allelic exchange screening
HC75	/	ggtggtaacacgtaagcaatgctttcgtcc	
HC115	ccatatgaccatgattacgaattcgagctc	CAGCAAGAGAGTTTTTCTATAAGC	
HC146	GGACGCGTGACGTCGACTCTAGAGGATCC	gggtctagacccaagtgctccag	
**Complementation plasmids**			
HC279	GAGCTCCTGCAGTAAAGGAGAAAATTTT	atgaaaattaaagataataaggctg	CD630_32570 (*pgdB*) complementation
HC280	GGATCCGGTGCGCCAGGAG	ttataagttttcttgccagaaatttttag	
HC273	GAGCTCCTGCAGTAAAGGAGAAAATTTT	atggtaaaaaaaagaaaaaagaaaactg	CD630_15220 (*pgdA*) complementation
HC274	GGATCCGGTGCGCCAGGAG	ttatattaaaattttaaattgataaccattgtc	
RPF185A	/	AAAATTTTCTCCTTTACTGCAGGAGCTC	
RPF185B	/	CTCCTGGCGCACCGGATCC	
**Promoter identification**			
HC49	/	tgcatgaaaactcaaataccataga	CD630_32570 (*pgdB*) promoter identification
HC50	/	tagtcctcatacccatcttttgaag	
HC51	/	aaaagccctgtattcaactatcatc	
HC52	/	tctgtatcttctgtcacatccaatc	
HC53	/	acgaatatggaaactccatttgata	
HC54	/	ccagtgtattttcaccttttgaact	

Table S4. Primers used in the study
